# Isolation and Structure Elucidation of Novel Mycosporine-like Amino Acids from the Two Intertidal Red Macroalgae *Bostrychia scorpioides* and *Catenella caespitosa*

**DOI:** 10.3390/md21100543

**Published:** 2023-10-18

**Authors:** Maria Orfanoudaki, Mostafa Alilou, Anja Hartmann, Julia Mayr, Ulf Karsten, Hieu Nguyen-Ngoc, Markus Ganzera

**Affiliations:** 1 Institute of Pharmacy, Pharmacognosy, University of Innsbruck, Innrain 80-82, 6020 Innsbruck, Austria; orfmaria@gmail.com (M.O.); mostafa.alilou@uibk.ac.at (M.A.); anja-k-hartmann@web.de (A.H.); julia_mayr1@gmx.at (J.M.); hieu.nguyenngoc@phenikaa-uni.edu.vn (H.N.-N.); 2 Institute of Biological Sciences, Applied Ecology & Phycology, University of Rostock, Albert-Einstein-Str. 3, 18059 Rostock, Germany; ulf.karsten@uni-rostock.de; 3 Faculty of Pharmacy, Phenikaa University, Hanoi 12116, Vietnam; 4 A&A Green Phoenix Group JSC, Phenikaa Research and Technology Institute (PRATI), No.167 Hoang Ngan, Trung Hoa, Cau Giay, Hanoi 11313, Vietnam

**Keywords:** *Bostrychia scorpioides*, *Catenella caespitosa*, mycosporine-like amino acids, bostrychines, catenellines

## Abstract

This study presents a phytochemical survey of two common intertidal red algal species, *Bostrychia scorpioides* and *Catenella caespitosa*, regarding their MAA (mycosporine-like amino acid) composition, which are known as biogenic sunscreen compounds. Six novel MAAs from *Bostrychia scorpioides* named bostrychines and two novel MAAs from *Catenella caespitosa* named catenellines were isolated using a protocol which included silica gel column chromatography, flash chromatography on reversed phase material and semipreparative HPLC (High-Performance Liquid Chromatography). The structure of the novel MAAs was elucidated using NMR (Nuclear Magnetic Resonance) and HR-MS (High-Resolution Mass Spectrometry), and their absolute configuration was confirmed by ECD (Electronic Circular Dichroism). All isolated MAAs possess a cyclohexenimine scaffold, and the metabolites from *B*. *scorpioides* are related to the known MAAs bostrychines A-F, which contain glutamine, glutamic acid and/or threonine in their side chains. The new MAAs from *C*. *caespitosa* contain taurine, an amino sulfonic acid that is also present in another MAA isolated from this species, namely, catenelline. Previous and new data confirm that intertidal red algae are chemically rich in MAAs, which explains their high tolerance against biologically harmful ultraviolet radiation.

## 1. Introduction

*Bostrychia scorpioides* (Hudson) Montagne ex Kützing and *Catenella caespitosa* (Withering) L. M. Irvine are two of the most common red macroalgae growing in the intertidal zone of estuarine to marine coasts in Europe [1].

*Bostrychia scorpioides* is the only species out of 40 accepted taxa of the genus *Bostrychia* abundant on European coasts [2,3]. It is an Atlantic estuarine and marine species living as epiphyte on salt marsh plants such as *Halimione portulacoides* in the upper intertidal from the British Isles to Morocco. Other *Bostrychia* species mainly occur as epiphytes on mangrove root systems in warm-temperate to tropical regions [4]. These algae tolerate long periods of desiccation during the tidal cycle due to their high-shore position [5], and they survive several weeks exposed to air by adjusting the intracellular solute content, resulting in partial or complete turgor regulation. This is achieved by changing internal concentrations of the main ions K^+^, Na^+^ and Cl^−^ and adopting in the organic osmolyte content such as polyols (sorbitol and dulcitol), amino acids or quaternary ammonium compounds [2]. Additionally, *B. scorpioides* produces a set of unique mycosporine-like amino acids (MAAs), bostrychines A to F (approximately 2–10 mg/g of dry weight in total) [6,7], compounds with established photoprotective but questionable osmoprotective properties [8]. Because of its high number of representatives of this compound class (eight to twelve MAAs), it stands out among other red algae of the same biogeographic region [9] or other *Bostrychia* species from all over the world. *B. scorpioides* has also been reported to produce a high number of the carotenoids violaxanthin, zeaxanthin and β-carotene (in total, 0.65 mg/g DW) and a significantly increased concentration of phycoerythrin and chlorophyll *a* in summer, as compared to in winter, possibly due to the higher exposure to light radiation and desiccation stress [9].

Only five species of the genus *Catenella* have been accepted taxonomically [3]. One of them, *C. caespitosa*, has a wide biogeographic distribution in Europe and America, and it grows on sheltered shady rocks or sediment between high tide levels and supralittoral zones [1,10]. Catenelline, a sulfonic acid-bearing MAA, has been isolated as the main MAA from the former species *Catenella repens* (synonym of *C. caespitosa)* with an amount ranging from 0.3–1.8 mg/g of dry weight, but at least one additional but unknown MAA is reported to be present in lower amounts [10].

The main focus of the present study was to complete the phytochemical profile of two intertidal red macroalgae found in Europe, *B. scorpioides* and *C. caespitosa*. Previous studies indicated that both produce MAAs, which had not been identified [6,10]. Thus, this study focused on the isolation of yet-unexplored UV-sunscreen molecules.

MAAs are water soluble, colourless secondary metabolites found in variety of marine and aquatic organisms including algae, bacteria, fungi, lichens, corals, sponges, sea urchins, scallop and fish [11,12,13] in order to mitigate the harmful effects of UV irradiation [14], as well as desiccation or osmotic stress [15]. They also serve as nitrogen reservoirs during nitrogen limitation [16]. They have a 5-hydroxy-5-hydroxymethyl-cyclohex-1, 2-ene ring structure and a methoxy-substituent in C2, and they are further substituted by a variety of amino acids or other substituents at positions 1 and 3 [14,17]. Some terrestrial organisms, e.g., the cyanobacterium *Nostoc commune* [18], green algae of the genera *Coelastrella* [19], *Interfilum* and *Klebsormidium* [20] and the terrestrial fungus *Pyronema omphalodes* [21] produce MAAs too, which are in most cases glycosylated [18,20,21]. Depending on the substitution at position 1, they can be divided into the cyclohexenone type, with an absorption maximum at 310 nm in the UV-B range, or the cyclohexenimine type, with a maximum in the UV-A region (320–360 nm) [14,18,20]. The have high molar absorption coefficients (ε = 12.400–58.800 M^−1^·cm^−1^) [22]. Their biosynthetic pathways include either the shikimate or pentose phosphate pathway [17].

MAAs are commercially attractive compounds since they are biocompatible, biodegradable and have no toxic properties [23,24]. Some commercial formulations such as Helioguard^®^365 and Helionori^®^ contain them as active ingredients [25]. They show a promising future for applications in the pharmaceutical and cosmetic industries as natural sunscreens and anti-photoaging molecules, being in demand because of the rapid increase in skin damage in humans due to UV radiation [26] and the harmful effects of synthetic sunscreens on human health and the environment [14,23]. Unfortunately, the utilization of their biological sources for the mass production of these economically important molecules has been hindered because of the incomplete understanding of their effect on human skin and their low abundance in the producing organisms [13,17]. Algaculture, biotechnology or semisynthetic approaches could be alternatives for overcoming the supply issue [17,27].

## 2. Results

### 2.1. Isolation

The combined aqueous and methanolic extracts of *B. scorpioides* and *C. caespitosa* were first fractionated on a silica gel column in order to remove non-polar constituents such as chlorophylls in the early eluting fractions and sugars in the later, more polar fractions so as to obtain MAA-enriched fractions (details in Section 4.2). This initial step was followed by separations using reversed phase material on flash chromatography and semipreparative HPLC. The purification procedure resulted in the isolation of six novel compounds from *B. scorpioides* and two novel compounds from *C. caespitosa.*

### 2.2. Structure Elucidation

Characteristic NMR (Nuclear Magnetic Resonance) shifts of compound **1** (Table 1 and Table 2) indicated the presence of a cyclohexenimine-type MAA scaffold. The side chain was identified as glutamine based on a COSY (Correlation Spectroscopy) chain correlation of H-9/H-11/H-12 and two carbonyl groups at *δ*_C_ 179.5 and 181.1 and by comparison with the literature values [6]. Its position was confirmed by long-range correlations visible in the HMBC (Heteronuclear Multiple Bond Correlation) spectrum (H-9 at *δ*_H_ 4.21 to C-3 at *δ*_C_ 161.7). Specifically, a methine group at *δ*_H_ 4.21 (H-9) showed a correlation in the COSY spectrum with the protons of the methylene at *δ*_H_ 2.18 and *δ*_H_ 2.27 (H-11) and an HMBC correlation with the carbonyl group at *δ*_C_ 179.5 (C-10). Furthermore, the protons of the methylene at C-12 (*δ*_H_ 2.45) showed a correlation in the COSY spectrum with the protons of the methylene at position 11 (*δ*_H_ 2.18 and 2.27) and an HMBC correlation with the carbonyl group at *δ*_C_ 181.1 (C-13). However, the ^1^H-NMR spectrum revealed extra signals, two doublets of doublets of a methylene at *δ*_H_ 3.44/3.51 (H-1′), a multiplet of an oxygenated methine at *δ*_H_ 4.03 (H-2′) and a doublet of a methyl-group at *δ*_H_ 1.24 (*J* = 6.4 Hz, H-3′), which showed a correlation in the COSY spectrum (H-1′/H-2′/H-3′). These signals, in addition to the HMBC connectivities (H-3′/C-2′ and C-1′, H-1′/C-2′ and C-3′), indicated the presence of a threamine moiety. The HMBC correlation of the same proton (H-1′) to carbon C-1 at *δ*_C_ 163.5 confirmed that threamine is attached to position C-1. The two amino acid residues of the side chains of this MAA have already been observed individually in other MAAs; glutamine has been found in MAAs from *B. scorpioides*—for example, in bostrychines A and B [6], while threamine is a constituent of the MAAs aplysiapalythine A and bostrychine E [6,28].

**Figure 1:**
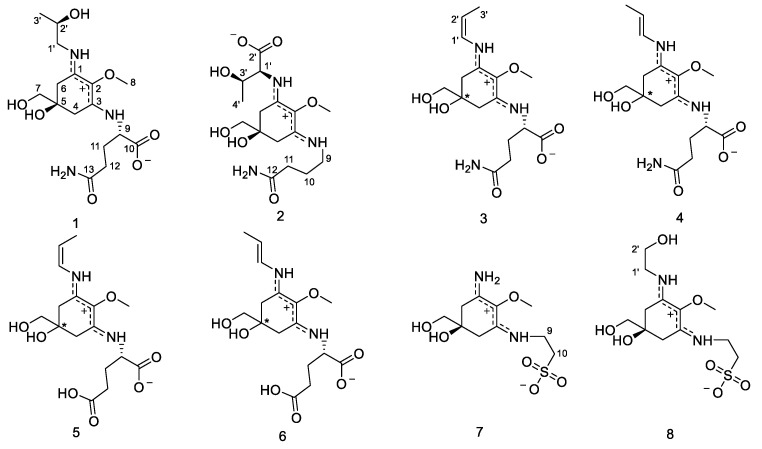
The structures of the novel MAAs from *Bostrychia scorpioides* (**1**–**6**) and *Catenella caespitosa* (**7**, **8**).

The advanced Marfey’s method, an established LC-MS (Liquid Chromatography–Mass Spectrometry)-based procedure for determining the absolute configuration, has confirmed the presence of l-glutamic acid and *R*-threamine as constituent of other MAAs in this species [7]. In order to determine the absolute stereochemistry of C-5, geometrical optimization followed by ECD (Electronic Circular Dichroism) calculation at the m062x/6−31 + g(d,p)/smd//wb97xd/6−31 + g(d,p) level in H_2_O resulted in an ECD spectrum showing a high similarity to the experimentally obtained spectrum. The absolute configuration was established as 5*R*,1′*S*,3′*R*, and therefore, compound **1** (Figure 1) was finally identified as a new MAA, (*S*)-5-amino-2-(((*S*,*E*)-5-hydroxy-5-(hydroxymethyl)-3-(((*R*)-2-hydroxypropyl)amino)-2-methoxycyclohex-2-en-1-ylidene)ammonio)-5-oxopentanoate, with the molecular formula C_16_H_27_N_3_O_7_ (high-resolution MS data: for [M + H]^+^, *m*/*z* = 374.1911; calculated for C_16_H_28_N_3_O_7_, 374.1927), for which we propose the trivial name bostrychine G. 

**Figure 2:**
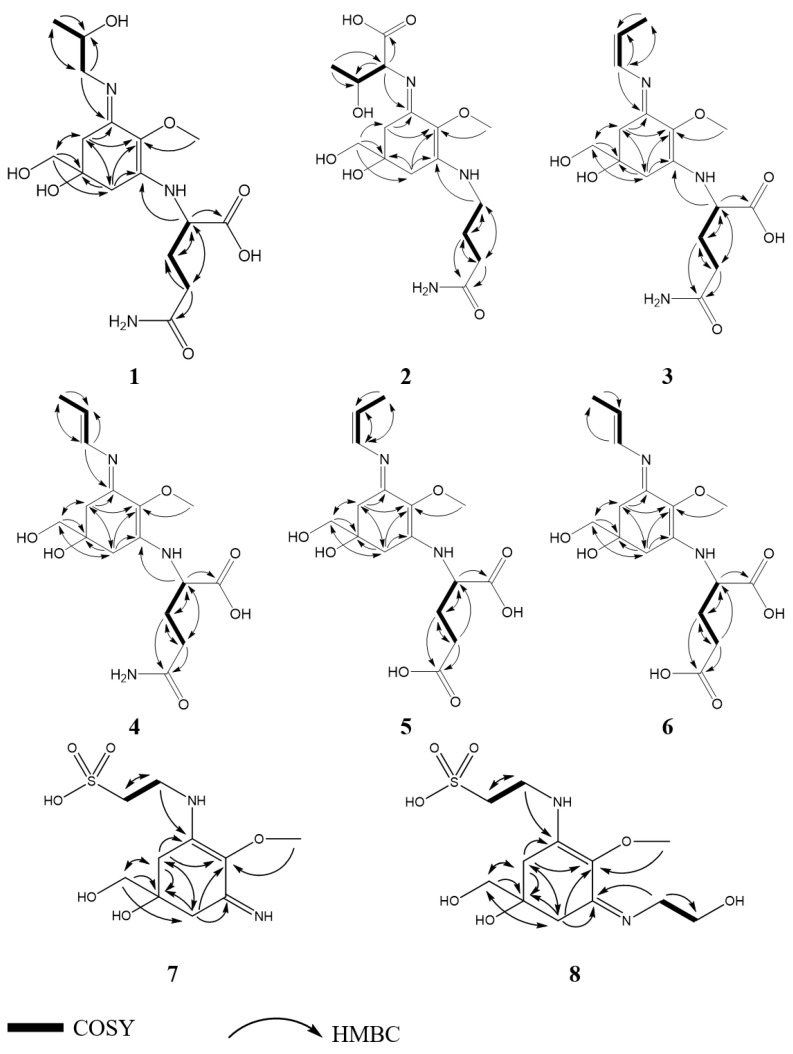
Key ^1^H-^13^C HMBC (indicated by arrows) and ^1^H-^1^H COSY correlations (indicated by bold bonds) of compounds **1**–**8** from *Bostrychia scorpioides* and *Catenella caespitosa.*

Compound **2** was assigned the molecular formula C_16_H_27_N_3_O_7_, as established by a positive ion (for [M + H]^+^, *m*/*z* = 374.1913; calculated for C_16_H_28_N_3_O_7_, 374.1927) in the HR-MS (High-Resolution–Mass Spectrometry) spectrum. Characteristic NMR chemical shifts indicated the presence of a cyclohexenimine-type MAA. The side chain was identified as threonine based on a COSY chain correlation of H-1′/H-3′/H-4′ and a carbonyl group at *δ*_C_ 178.3 and by comparison with the literature values. Its position was confirmed by long-range correlations visible in the HMBC spectrum (H-1′ at *δ*_H_ 4.05 to C-3 at *δ*_C_ 162.2). The COSY spectrum revealed an extra coupling network, including the protons of three methylene units H-9/H-10/H-11. Additionally, HMBC correlations of H-10 (*δ*_H_ 1.96) and H-11 (*δ*_H_ 2.39) to a carbonyl group at *δ*_C_ 180.7 (C-12) revealed the presence of 4-aminobutanamide as a side chain (Figure 2). An HMBC correlation of proton H-9 (*δ*_H_ 3.51) to carbon C-1 at *δ*_C_ 163.4 indicated at which position the 4-aminobutanamide moiety is attached to the cyclohexenimine scaffold. The advanced Marfey’s method of bostrychines B, D and F has shown the presence of l-threonine as a constituent of the final product of the reaction, indicating that this amino acid residue in the MAA has an l-configuration [7]. The determination of the absolute stereochemistry of compound **2** by geometrical optimization followed by ECD calculation at the m062x/6−31 + g(d,p)/smd//wb97xd/6−31 + g(d,p) level in H_2_O resulted in an ECD spectrum showing an excellent match with the experimentally obtained spectrum, therefore establishing the absolute configuration as 5*R*,1′*S*,3′*R*. Compound **2** was finally identified as a new MAA, (2*S*,3*R*)-2-(((*R*,1*E*,3*E*)-3-((4-amino-4-oxobutyl)-l4-azaneylidene)-5-hydroxy-5-(hydroxymethyl)-2-methoxycyclohexan-2-ylium-1-ylidene)-l4-azaneyl)-3-hydroxybutanoate, for which we propose the trivial name bostrychine H.

**Figure 3:**
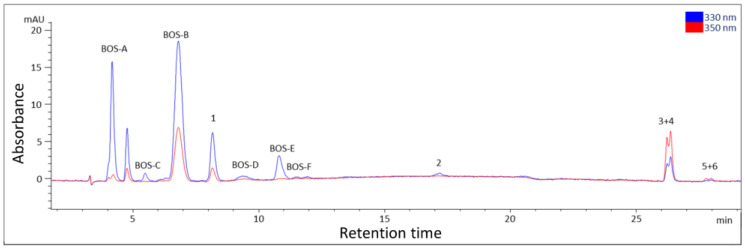
High-Performance Liquid Chromatography–Ultraviolet (HPLC-UV) separation of the *Bostrychia scorpioides* extract. Peak assignment is according to Figure 1; BOS-A: bostrychine-A, BOS-B: bostrychine-B, BOS-C: bostrychine-C, BOS-D: bostrychine-D, BOS-E: bostrychine-E, BOS-F: bostrychine-F; column: Pack Pro C18 RS (150 × 4.6 mm, 3 µm) from YMC; mobile phase: 0.9% (*v*/*v*) formic acid and 0.1% (*v*/*v*) acetic acid in water (A) and methanol (B); gradient: 0–15 min: 0% B, 23 min: 10% B, 30 min: 15% B, 35–40 min: 98% B, 40.1–55 min: 0% B; λ = 330 and 350 nm; flow rate = 0.55 mL/min; T = 25 °C.

Compounds **3** and **4** had similar retention times in the HPLC chromatogram (Figure 3), and they were obtained as a mixture. The integration of the signals in the ^1^H-NMR spectra showed that the relative ratio of the two compounds was 0.7:1 (compound **3**: compound **4**). The side chain of both compounds at position 3 was identified as glutamine based on COSY chain correlations of H-9/H-11/H-12 and characteristic NMR values which were in accordance with the literature values. The second side chain of compound **3** contained a *cis* conformation double bond, which was indicated by characteristic ^1^H-NMR (H-1′, *δ*_H_ 6.39 and H-2′, *δ*_H_ 5.42) and ^13^C-NMR (C-1′, *δ*_C_ 124.7 and C-2′, *δ*_C_ 120.3) values and the coupling constants of H-1′ (*J* = 8.0/1.2 Hz). Moreover, a methyl group at *δ*_H_ 1.78 (H-3′) showed a correlation in the COSY spectrum with the protons of H-2′ at *δ*_H_ 5.42 and an HMBC correlation with the carbons of the double bond at *δ*_C_ 120.3 and 124.7 (C-2′ and C-1′), revealing the presence of a propene side group. Its position was confirmed by long-range correlations visible in the HMBC spectrum (H-1′ at *δ*_H_ 6.39 to C-1 at *δ*_C_ 158.0). The NMR signals of compound **4** were highly similar to those of compound **3**. However, protons H-1′ and H-2′ were deshielded (H-1′, *δ*_H_ 6.57 and H-2′, *δ*_H_ 5.77), and the coupling constants of H-1′ were higher (*J* = 13.6/2.0 Hz), indicating that the conformation of the double bond was *trans*. Thus, compounds **3** and **4** were identified as (2*S*)-5-amino-2-(((1*E*,3*E*)-5-hydroxy-5-(hydroxymethyl)-2-methoxy-3-(((*Z*)-prop-1-en-1-yl)-l4-azaneylidene)cyclohexan-2-ylium-1-ylidene)-l4-azaneyl)-5-oxopentanoate and (2*S*)-5-amino-2-(((1*E*,3*E*)-5-hydroxy-5-(hydroxymethyl)-2-methoxy-3-(((*E*)-prop-1-en-1-yl)-l4-azaneylidene)cyclohexan-2-ylium-1-ylidene)-l4-azaneyl)-5-oxopentanoate, respectively. They are new MAAs with the molecular formula C_16_H_25_N_3_O_6_ (high-resolution MS data: [M + H] ^+^ = 356.1808; calculated for C_16_H_26_N_3_O_6_, 356.1822, Figure S20) and the proposed trivial names bostrychines I and J, respectively. 

Compounds **5** and **6** were obtained as a mixture, and the integration of the signals in the ^1^H-NMR spectrum indicated that they were present in almost equal concentrations. Additionally, the NMR shifts were highly similar to those of compounds **3** and **4**, with the exception of the carbon atoms C-12 and C-13, which were slightly deshielded, indicating the presence of a carboxylic acid at position C-13 instead of an amide functionality. Furthermore, the HRMS data indicated the molecular formula C_16_H_24_N_2_O_7_ (for [M + H]^+^, *m*/*z* = 357.1648; calculated for C_16_H_25_N_2_O_7_, 357.1662), which confirmed the presence of glutamic acid at C-3 of the cyclohexenimine structure. Compounds **5** and **6** were finally identified as (2*S*)-4-carboxy-2-(((1*E*,3*E*)-5-hydroxy-5-(hydroxymethyl)-2-methoxy-3-(((*Z*)-prop-1-en-1-yl)-l4-azaneylidene)cyclohexan-2-ylium-1-ylidene)-l4-azaneyl)butanoate and (2*S*)-4-carboxy-2-(((1*E*,3*E*)-5-hydroxy-5-(hydroxymethyl)-2-methoxy-3-(((*E*)-prop-1-en-1-yl)-l4-azaneylidene)cyclohexan-2-ylium-1-ylidene)-l4-azaneyl)butanoate, for which we propose the trivial names bostrychines K and L.

**Figure 4:**
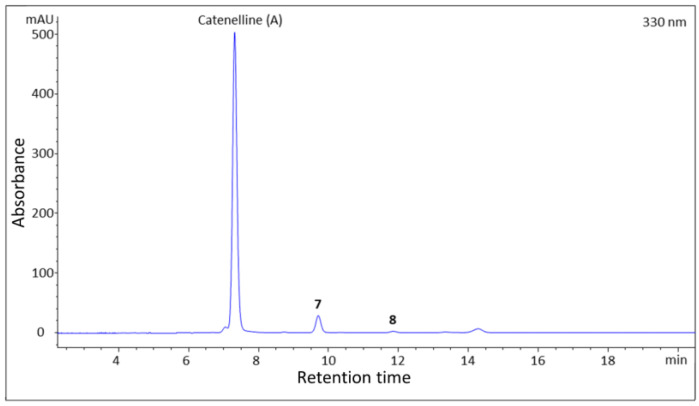
High-Performance Liquid Chromatography–Ultraviolet (HPLC-UV) separation of the *Catenella caespitosa* extract. Peak assignment is according to Figure 1; **7**: Catenelline B, **8**: Catenelline C; column: Pack ODS (250 × 4.60 mm, 5 μm) from YMC; mobile phase: 20 mM ammonium formate and 0.25% (*v*/*v*) formic acid in water (A) and methanol (B); gradient: 0–20 min: 0% B, 30 min: 20% B, 35–40 min: 98% B, 40.1–55 min: 0% B; λ = 330 nm; flow rate = 0.65 mL/min; T = 7 °C.

**Figure 5:**
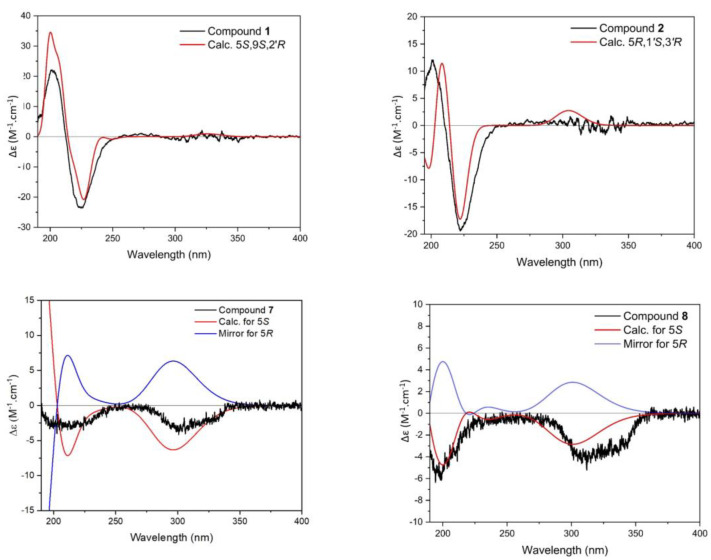
Electronic Circular Dichroism spectra (calculated: red; calculated mirror image: blue) of compounds **1**, **2** (*Bostrychia scorpioides*) and **7**, **8** (*Catenella caespitosa*) compared to their experimentally recorded spectra (black curves) in H_2_O. Δε is the molar ellipticity of the compounds, “Calc.” is the abbreviation for “calculated for” and mirror displays the opposite spectra corresponding to the respective enantiomer of each compound.

When analyzing the *C. caespitosa* extract by HPLC, compound **7** eluted after catenelline (Figure 4), and characteristic NMR shifts indicated the presence of a cyclohexenimine-type MAA, too. The side chain was identified as 2-aminoethane-1-sulfonic acid based on a COSY chain correlation of H-9/H-10 and by comparison with the literature values [9]. Its position was evidenced by long-range correlations visible in the HMBC spectrum (H-9 at *δ*_H_ 3.87 to C-3 at *δ*_C_ 163.3), and relevant connectivities are indicated by arrows in Figure 2. Specifically, a methylene group at *δ*_H_ 3.87 (H-9) showed a correlation in the COSY spectrum with the protons of the methylene at *δ*_H_ 3.23 (H-10), as well as a HMBC correlation with the carbon at *δ*_C_ 42.1 (C-10). 2-aminoethane-1-sulfonate has already been found as a moiety in catenelline. The latter was first described by Hartmann et al. in 2015 [12]. A comparison of the experimental and calculated spectrum at the m062x/6−31 + g(d,p)/smd//wb97xd/6−31 + g(d,p) level in the H_2_O of compound **7** (Figure 5) indicated the *S* chirality of C-5. Compound **7** was finally identified as a new MAA, (*S*,*Z*)-2-((3-(l4-azaneylidene)-5-hydroxy-5-(hydroxymethyl)-2-methoxycyclohexan-2-ylium-1-ylidene)-l4-azaneyl)ethane-1-sulfonate, with the molecular formula: C_10_H_18_N_2_O_6_S (high-resolution MS data: for [M + H]^+^, *m*/*z* = 295.0947; calculated for C_10_H_19_N_2_O_6_S, 295.0964; Figure S32) and the proposed trivial name catenelline B. 

The characteristic NMR shifts (Table 1 and Table 2) and 2D-NMR data of compound **8** indicated the same substructure as that of compound **7**. However, the ^1^H-NMR spectrum showed two additional signals, two triplets at *δ*_H_ 3.60 (*J* 5.4 Hz, H-11) and *δ*_H_ 3.77 (*J* = 5.4 Hz, H-12). The protons of these two methylene groups showed a correlation in the COSY spectrum, and in the HMBC, the protons of H-11 showed a correlation with the carbon at *δ*_C_ 63.1 (C-12), revealing the presence of a 2-aminoethan-1-ol moiety. Furthermore, the HMBC correlation of the same proton (H-11) to carbon C-3 at *δ*_C_ 163.3 indicated that this side chain is attached to position C-3. The purity of this compound was approximately 70%; thus, the optical rotation value and the molar absorption coefficient (ε) were not determined. The ECD spectrum of compound **8** was highly similar to that of compound **7**. The calculation of the ECD spectrum at the m062x/6−31 + g(d,p)/smd//wb97xd/6−31 + g(d,p) level in H_2_O and the comparison with the experimental spectrum in H_2_O resulted in deciphering the absolute stereochemistry as 5*S*. As the NMR data of all substructures were in good agreement with the literature values, compound **8** was finally identified as a new MAA, 2-(((*S*,1*Z*,3*E*)-5-hydroxy-3-((2-hydroxyethyl)-l4-azaneylidene)-5-(hydroxymethyl)-2-methoxycyclohexan-2-ylium-1-ylidene)-l4-azaneyl)ethane-1-sulfonate, with the molecular formula: C_12_H_22_N_2_O_7_S (high-resolution MS data: for [M + H]^+^, *m*/*z* = 339.1212; calculated for C_12_H_23_N_2_O_7_S, 339.1226), for which we propose the trivial name catenelline C.

### 2.3. Physical and Spectroscopic Data

#### 2.3.1. Compound **1**

Pale yellow amorphous powder; [α]^21^_D_ = −14.8 (c 1, H_2_O); UV λ_max_ = 334 nm; ε = 28,535 M^−1^·cm^−1^; ^1^H and ^13^C NMR data (600/151 MHz; D_2_O), Table 1 and Table 2; ESIMS: [M + H]^+^, *m*/*z* = 374; HRESIMS: [M + H]^+^, *m*/*z* = 374.1911; calculated for C_16_H_28_N_3_O_7_, 374.1927).

#### 2.3.2. Compound **2**

Pale yellow amorphous powder; [α]^21^_D_ = −10.2 (c 1, H_2_O); UV λ_max_ = 334 nm; ε = 19,571 M^−1^·cm^−1^; ^1^H and ^13^C NMR data (600/151 MHz; D_2_O), Table 1 and Table 2; ESIMS: [M + H]^+^, *m*/*z* = 374; HRESIMS: [M + H]^+^, *m*/*z* = 374.1913 (calculated for C_16_H_28_N_3_O_7_, 374.1927).

#### 2.3.3. Compound **3**

Pale yellow amorphous powder; UV λ_max_ = 360 nm; ^1^H and ^13^C NMR data (600/151 MHz; D_2_O), Table 1 and Table 2; ESIMS: [M + H]^+^, *m*/*z* = 356; HRESIMS: [M + H]^+^, *m*/*z* = 356.1808 (calculated for C_16_H_26_N_3_O_6_, 356.1822).

#### 2.3.4. Compound **4**

Pale yellow amorphous powder; UV λ_max_ = 360 nm; ^1^H and ^13^C NMR data (600/151 MHz; D_2_O), Table 1 and Table 2 ESIMS: [M + H]^+^, *m*/*z* = 356; HRESIMS: [M + H]^+^, *m*/*z* = 356.1808 (calculated for C_16_H_26_N_3_O_6_, 356.1822).

#### 2.3.5. Compound **5**

Pale yellow amorphous powder; UV λ_max_ = 357 nm; ^1^H and ^13^C NMR data (600/151 MHz; D_2_O), Table 1 and Table 2; ESIMS *m*/*z* 357 [M + H]^+^; HRESIMS: *m*/*z* 357.1648 [M + H]^+^ (calculated for C_16_H_25_N_2_O_7_, 357.1662).

#### 2.3.6. Compound **6**

Pale yellow amorphous powder; UV λ_max_ = 357 nm; ^1^H and ^13^C NMR data (600/151 MHz; D_2_O), Table 1 and Table 2; ESIMS: [M + H]^+^, *m*/*z* = 357; HRESIMS: [M + H]^+^, *m*/*z* = 357.1648 (calculated for C_16_H_25_N_2_O_7_, 357.1662).

#### 2.3.7. Compound **7**

Pale yellow amorphous powder; [α]^21^_D_ = +59.7 (c 1, H_2_O); UV λ_max_ = 320 nm; ε = 73,825 M^−1^·cm^−1^; ^1^H and ^13^C NMR data (600/151 MHz; D_2_O), Table 1 and Table 2; ESIMS: [M + H]^+^, *m*/*z* = 295; HRESIMS: [M + H]^+^, *m*/*z* = 295.0947 (calculated for C_10_H_19_N_2_O_6_S, 295.0964).

#### 2.3.8. Compound **8**

Pale yellow amorphous powder; UV λ_max_ = 330 nm; ^1^H and ^13^C NMR data (600/151 MHz; D_2_O), Table 1 and Table 2; ESIMS: [M + H]^+^, *m*/*z* = 339; HRESIMS: [M + H]^+^, *m*/*z* = 339.1212 (calculated for C_12_H_23_N_2_O_7_S, 339.1226).

## 3. Discussion

Mycosporine-like amino acids are compounds with photoprotective, antioxidant [22], anti-inflammatory [29], wound-healing [30] and immunomodulatory [31] properties. The producing organisms are mainly various micro- and macroalgae, and the prevailing hypothesis is that they synthesize MAAs because they protect against the UV damages of sun irradiation and thermal stress [32]. They might also participate in the osmotic equilibrium, especially in photosymbiotic partnerships [33]. The osmotic function of MAAs in marine organisms, however, has been questioned because the intracellular concentrations are generally too low for a major contribution to the osmotic potential [34]. MAAs also have other physiological functions; they serve as antioxidants [32], ROS-scavengers [35,36], reproductive regulators [37] and an intracellular nitrogen reservoir [17], while some studies suggest that MAAs, especially in cyanobacteria, play a negligible role in the protection against UV radiation and might be involved in the formation of extracellular matrix and cell–cell interactions [38,39,40].

Recently, MAAs have attracted increasing attention, and there are a large number of patents in international databases for several products and methods with MAAs [22,29]. They offer a great potential for the development of novel UV sunscreens because of their direct and indirect protective properties. Therefore, the investigation of unexplored MAA-producing organisms and their unambiguous structure elucidation increase the understanding on chemical diversity [29]. It is noteworthy to mention that most of the MAAs utilized in these patents provide minimal protection in the more damaging UVB range. Additionally, the MAA concentration in the algal extract is often very low when compared to the concentration of other photoprotective ingredients in most sunscreen products [22,41]; thus, the identification of organisms producing higher yields of these metabolites or the preparation of semisynthetic analogues is very important.

In the present study, MAAs of two intertidal red macroalgae, *B. scorpioides* and C*. caespitose,* were investigated. The presence of these MAAs had been described in previous studies, although their isolation and structural assignment were not feasible previously [6,9,10]. After the usage of a slightly different isolation protocol in this study, six novel MAAs were obtained from *B. scorpioides* and two novel compounds were obtained from *C. caespitosa* using chromatographic techniques. Their structures were elucidated, and their stereochemistry was revealed after the combination of NMR and ECD experiments. 

The newly isolated MAAs from *B. scorpioides* are related to the reported bostrychines A-F [6] since they contain the amino acid residues threonine, threamine, glutamine and glutamic acid in their side chains. Compounds **3**–**6** (bostrychines I to L) additionally contain (*E*)- and (*Z*)-prop-1-en-1-amine, which is also a substituent of the known MAAs usujirene [42] and palythene [43] and another MAA tentatively identified as dehydroxyl-usujirene [44]. It is notable that the latter three MAAs, along with the newly isolated bostrychines I-L, all containing an extra double bond in their side chain, have a λ_max_ of 357–360 nm in water. This is uncommon since the majority of cyclohexenimine-type MAAs have an λ_max_ of 325–337 nm in the same solvent, while those with a cyclohexenone scaffold show a maximum absorption of around 310–324 nm in water [22,33,45]. Regarding the MAAs isolated from *C. caespitosa*, both compounds contained taurine in their side chain, which is also reported to be a moiety of catenelline, isolated from the same species previously [10]. Additionally, there is a high similarity of catenelline B and mycosporine-taurine isolated from the sea anemone *Anthopleura elegantissima*, with the difference being that the first MAA possesses the cyclohexenimine scaffold (λ_max_: 320 nm in water), whereas the second one possesses the cyclohexenone scaffold (λ_max_: 309 nm) [46].

*Bostrychia scorpioides* is an ecologically fascinating red alga, as it grows as an epiphyte on the basal stems of saltmarsh vegetation. Consequently, it is immersed by seawater only during extreme high tides and hence exhibits a rather atmophytic lifestyle due to long periods of exposure to air, leading to enhanced desiccation and osmotic and radiation stress [6]. These stressors can be well compensated by various ecophysiological and biochemical traits. Most important is the capability to synthesize and accumulate protective organic compounds like the polyols sorbitol and dulcitol [2]. In addition, *B. scorpioides* is regularly confronted with high solar insolation including ultraviolet radiation (UVR). UVR affects various biological functions in living organisms, and extensive exposure can lead to significant stress and deleterious effects at the molecular and cellular level [47,48,49]. Adaptive mechanisms against enhanced UVR typically include in many micro- and macroalgae photoprotective MAAs [50]. Most algae investigated so far typically contain a small set of MAAs (one to five) [9,51], while *B. scorpioides* exhibited at least twelve chemically different MAAs, named in a previous publication and in the present publication as bostrychines A to L [6,7]. The biosynthesis of each of these MAAs requires individual enzymatic steps [8,52], and hence, the question arises of why this red alga invests so much metabolic energy for the formation of such an array of chemically similar compounds. As we can only speculate at this stage, it might be possible that bostrychines A to L all exhibit slightly different functions as anti-stress compounds, i.e., some act as UV-sunscreens and others as antioxidants.

*Catenella caespitosa* also grows as an intertidal species mainly exposed to the atmosphere. Although it can occur as epiphyte on saltmarsh plants, too, this species preferentially grows in the upper littoral zone on exposed rocky shores. For salinity acclimation, *C. caespitosa* uses as an organic osmolyte floridoside, and for UVR protection, it uses the previously identified MAA catenelline [10]. In the present study, two additional and chemically related MAAs (catenelline B and C) were identified, which might also be involved in the protection against UVR and oxygen radicals. 

## 4. Materials and Methods

### 4.1. Biological Material

The algae investigated in this study were collected in 2018 next to Roscoff, Brittany, France and morphologically identified by some of the authors (Maria Orfanoudaki, Anja Hartmann and Markus Ganzera), together with Ulf Karsten, from the University of Rostock, Germany, using their taxonomic expert knowledge in conjunction with standard identification keys [3,53]. Voucher samples were deposited at the Institute of Pharmacy, Pharmacognosy, University of Innsbruck, Austria (*B. scorpioides*) and at the University of Rostock, Germany (*C. caespitosa*).

### 4.2. Extraction and Isolation

The combined aqueous and methanolic extract of *B. scorpioides* was first fractionated on a silica gel column, followed by further purification using flash chromatography on reversed phase material and semipreparative HPLC. The procedure resulted in the isolation of six compounds. The same extraction and isolation protocol was used for the isolation and identification of MAAs from *C. caespitosa*, with the addition of column chromatography on Sephadex LH-20 as the last purification step.

*B. scorpioides* (approximately 350 g) was extracted three times in an ultrasonic bath (Bandelin Sonorex 35 KHz, Berlin, Germany) for 15 min successively with methanol and water (100%). Afterwards, the combined and dried extract (50 g) was fractionated on a silica gel column using EtOAc and methanol (from 10:0 to 0:10) as the eluent, resulting in 15 subfractions, in order to remove non-polar constituents such as chlorophylls in the early eluting fractions and sugars in the polar fractions. The HPLC analysis of the fractions indicated that fractions 9–12 contained MAAs; therefore, they were combined and used for further purification. This collective fraction (approximately 15 g) was separated with reversed phase flash chromatography using water–methanol (10:0–0:10) to give ten subfractions. Subfraction 6 (5 g) was subjected to semipreparative HPLC (H_2_O-MeOH) to yield compounds **1** (2 mg, 0.0006% yield) and **2** (2 mg, 0.0006% yield). Subfraction 7 (4 g) was also separated by semipreparative HPLC (H_2_O-MeOH) to give a mixture of compounds **3** and **4** (9.2 mg, 0.003% yield) and a mixture of compounds **5** and **6** (0.9 mg, 0.0003% yield). 

*C. caespitosa* (approximately 500 g) was extracted as described above. The combined methanolic and water extract (40 g) was fractionated on a silica gel column using EtOAc and methanol (from 10:0 to 0:10), resulting in 19 subfractions. The HPLC analysis of the fractions indicated that fractions 9–11 contained MAAs; therefore, they were pooled and used for further fractionation. The combined fractions 9–11 (approximately 12 g) were separated with reversed phase flash chromatography using water–methanol (10:0–0:10) to give 7 subfractions. The further purification of subfraction 2 (2 g) via semipreparative HPLC (H_2_O acidified with 0.1% formic acid-MeOH) and column chromatography on Sephadex LH-20 with methanol–water (3:1) resulted in the isolation of compounds **7** (8.8 mg, 0.002% yield) and **8** (1.1 mg, 0.0002% yield). 

### 4.3. Instrumentation

#### 4.3.1. Nuclear Magnetic Resonance 

NMR experiments were performed on a Bruker Avance II 600 spectrometer (Karlsruhe, Germany) operating at 600.19 (^1^H) and 150.91 MHz (^13^C). The isolated compounds were dissolved in D_2_O using tetramethylsilane (TMS) as an internal standard.

#### 4.3.2. Mass Spectrometry 

High-resolution mass spectra were measured with an Exploris 120 Orbitrap mass spectrometer (Thermo, MA, USA). The experiments were performed in positive ESI mode with the following parameters: capillary energy: 3500 V; sheath gas: 2 U; aux gas: 2 U and a vaporizer temperature of 250 °C; the recorded scan range was 100–600 *m*/*z* and the Orbitrap resolution was set to 15,000. Low-resolution mass spectra were recorded on an Agilent InfinityLab LC/MSD System. It comprised an Agilent 1260 HPLC, equipped with a binary pump, autosampler, column oven and photodiode array detector (Santa Clara, CA, USA). 

#### 4.3.3. Other Techniques Utilized

Optical rotations were measured with a P-2000 polarimeter (JASCO, Tokyo, Japan) using a 10.0 cm tube and water as the solvent. ECD experiments were conducted on a J-1500 spectrophotometer (JASCO, Tokyo, Japan). For the purification of compounds, a Reveleris^®^ X2 iES flash chromatography system (Büchi, Flawil, Switzerland) and a semi-preparative UltiMate 3000 HPLC from Dionex (Thermo, Waltham, MA, USA), comprising a P580 pump, an ASI 100 automated sample injector, an UVD 170 U detector and a fraction collector, were used. Sephadex LH-20 material was purchased from Sigma-Aldrich (St. Louis, MI, USA). Analytical HPLC experiments were performed on an LC-20AD XR System (Shimadzu, Tokyo, Japan). UV spectra and molar absorption coefficients were measured with a Shimadzu UV 1800 instrument after the dissolution in water, the blank (water) measurement, the measurement of the absorbance at λ_max_ and the conversion to ε according to the Beer–Lambert law.

### 4.4. Calculation of Electronic Circular Dichroism Spectra and Optical Rotation Calculation

3D structures of isolated compounds were drawn in Maestro (Schrödinger. LLC, New York, NY, USA) and subjected to conformational analysis using MacroModel 9.1 (Schrödinger. LLC) and OPLS-3 as a force field in water by the implementation of the Monte Carlo method. For the geometrical optimization and energy calculation of conformers occurring in an energy window of 2 Kcal·mol^−1^, the same methods as those in our previous publication on MAAs were utilized [30]. Briefly, geometry optimizations were conducted at DFT/wb97xd/6−31 + g(d,p) in the gas phase, followed by the calculation of ECD spectra at the TD--DFT/wb97xd/6−31 + g(d,p) level using SMD (Solvation Model Density) in water, using Gaussian 16 (Revision A.03, Gaussian, Wallingford, CT, USA 2016). The obtained ECD spectra (with a half-band of 0.25–0.3 eV and a UV shift of 8–20 nm) were Boltzmann-averaged and scaled spectra compared with the experimental spectra obtained in water.

### 4.5. Chemicals 

All of the solvents required for the extraction and isolation were purchased from VWR International (Vienna, Austria), and ethyl acetate (EtOAc) was distilled before use. The solvents for the analytical experiments a had pro analysis (p.a.) quality at least and were obtained from Merck (Darmstadt, Germany). Deuterated solvents were supplied by Euriso-Top (Saint-Aubin, France). Ultrapure water was produced by a Sartorius Arium^®^ 611 UV (Göttingen, Germany) purification system. Silica gel 40–63 µm and pre-packed cartridges for flash chromatography were purchased from Merck (Darmstadt, Germany) and Büchi (Flawil, Switzerland), respectively.

## Figures and Tables

**Table 1:** ^1^H NMR (Nuclear Magnetic Resonance) data of compounds **1**–**6** (isolated from *B. scorpioides*) and **7** and **8** (isolated from *Catenella caespitosa*).

pos.	1 (400 MHz)	2 (600 MHz)	3 (400 MHz)	4 (400 MHz)	5 (600 MHz)	6 (600 MHz)	7 (600 MHz)	8 (600 MHz)
	*δ*_H_ (*J* in Hz)	*δ*_H_ (*J* in Hz)	*δ*_H_ (*J* in Hz)	*δ*_H_ (*J* in Hz)	*δ*_H_ (*J* in Hz)	*δ*_H_ (*J* in Hz)	*δ*_H_ (*J* in Hz)	*δ*_H_ (*J* in Hz)
**4**	2.81, d (17.2) 2.76, d (17.2)	2.75, d (17.6) 2.90, d (17.6)	2.79–2.93 ^a^, d (18.0)	2.79–2.93 ^a^, d (18.0)	2.75–2.92 ^a^, d (18.0)	2.75–2.92 ^a^, d (18.0)	2.92, d (16.8) 2.98, d (16.8)	2.90, d (17.4) 2.92, d (17.4)
**6**	2.91, s	2.88, d (17.2) 2.92, d (17.2)	2.79–2.93 ^a^, d (18.0)	2.79–2.93 ^a^, d (18.0)	2.75–2.92 ^a^, d (18.0)	2.75–2.92 ^a^, d (18.0)	2.68, d (16.8) 2.94, d (16.8)	2.92, d (17.4) 2.97, d (17.4)
**7**	3.60, s	3.60, s	3.60, s	3.60, s	3.59, s	3.59, s	3.59, s	3.62, s
**8**	3.64, s	3.66, s	3.71, s	3.64, s	3.68–3.70 ^a^, s	3.68–3.70 ^a^, s	3.61, s	3.60, s
**9**	4.21, dd (8.0/4.8)	3.51, t (7. 2)	4.28, dd (8.0/4.8)	4.25, dd (8.0/4.8)	4.23–4.26 ^a^, dd (8.0/5.6)	4.23–4.26 ^a^, dd (8.0/5.6)	3.87, t (6.4)	3.87, td (6.0/1.2)
**10**		1.96, m					3.23, t (6.4)	3.23, t (6.0)
**11**	2.18, m 2.27, m	2.39, t (7.6)	2.20, m 2.28, m	2.20, m 2.28, m	2.13, m 2.25, m	2.13, m 2.25, m		
**12**	2.45, (td, 7.2/1.6)		2.46, m	2.46, m	2.35, m	2.35, m		
**1′**	3.44, dd (14.4/7.8) 3.51, dd (14.4/2.4)	4.05, d (3.6)	6.39, dd (8.0/1.2)	6.57, dd (13.6/2.0)	6.39, d (7.4)	6.56, d (15.0)		3.60, t (5.4)
**2′**	4.03, m		5.42, m	5.77, m	5.40, m	5.74, m		3.77, t (5.4)
**3′**	1.24, d (6.4)	4.31, m	1.78, dd (5.0/2.0)	1.76, dd (5.2/1.6)	1.76–1.77 ^a^, dd (6.6/1.8)	1.76–1.77 ^a^, dd (6.6/1.8)		
**4′**		1.26, d (6.4)						

^a^ overlapping signals.

**Table 2:** ^13^C NMR (Nuclear Magnetic Resonance) data of compounds **1–6** (isolated from *B. scorpioides*) and **7** and **8** (isolated from *Catenella caespitosa*).

pos.	1 ^d^	2 ^d^	3 ^d^	4 ^d^	5 ^e^	6 ^e^	7 ^e^	8 ^e^
	*δ*_C_, Type	*δ*_C_, Type	*δ*_C_, Type	*δ*_C_, Type	*δ*_C_, Type	*δ*_C_, Type	*δ*_C_, Type	*δ*_C_, Type
**1**	163.5, C	163.4, C	158.0, C	156.6, C	157.1, C^c^	157.1, C ^c^	163.3, C	162.0, C
**2**	128.5, C	128.3, C	129.1, C	128.6, C	128.7, C ^c^	128.7, C ^c^	127.5, C	128.2, C
**3**	161.7, C	162.2, C	163.8, C	162.8, C	163.8, C ^c^	163.8, C ^c^	164.3, C	163.3, C
**4**	35.9, CH_2_	35.5, CH_2_	35.8–36.1 ^a^, CH_2_	35.8–36.1 ^a^, CH_2_	35.7, CH_2_^b^	35.7, CH_2_^b^	36.1, CH_2_	35.8, CH_2_
**5**	73.9, C	73.6, C	73.8–73.9 ^a^, C	73.8–73.9 ^a^, C	73.9, C	73.9, C	74.2, C	73.9, C
**6**	35.9, CH_2_	36.1, CH_2_	35.8–36.1 ^a^, CH_2_	35.8–36.1 ^a^, CH_2_	35.7, CH_2_^b^	35.7, CH _2_^b^	38.6, CH_2_	35.4, CH_2_
**7**	70.5, CH_2_	70.3, CH_2_	70.4, CH_2_	70.4, CH_2_	70.5, CH_2_	70.5, CH_2_	70.3, CH_2_	70.3, CH_2_
**8**	62.1, CH_3_	62.2, CH_3_	62.3, CH_3_	62.4, CH_3_	62.4, CH_3_	62.4, CH_3_	61.9, CH_3_	61.9, CH_3_
**9**	61.3, CH	45.7, CH_2_	61.5–61.6 ^a^, CH	61.5–61.6 ^a^, CH	62.3, CH	62.3, CH	42.1, CH_2_	41.9, CH_2_
**10**	179.5, CO	28.0, CH_2_	178.9, CO	179.2, CO	179.6, CO ^c^	179.6, CO ^c^	52.5, CH_2_	52.6, CH_2_
**11**	30.6, CH_2_	34.8, CH_2_	30.4–30.5 ^a^, CH_2_	30.4–30.5 ^a^, CH_2_	31.0, CH_2_	31.0, CH_2_		
**12**	34.2, CH_2_	180.7, CO	34.2, CH_2_	34.2, CH_2_	36.7, CH_2_	36.7, CH_2_		
**13**	181.1, CO		181.1, CO	181.1, CO	184.5, CO ^c^	184.5, CO ^c^		
**1′**	52.9, CH_2_	67.2, CH	124.7, CH	126.5, CH	124.1, CH ^b^	126.0, CH ^b^		48.2, CH_2_
**2′**	69.5, CH	178.3, CO	120.3, CH	120.4, CH	119.5, CH ^b^	119.7, CH ^b^		63.1, CH_2_
**3′**	22.2, CH_3_	71.0, CH	13.5, CH_3_	17.3, CH_3_	13.4, CH_3_	17.3, CH_3_		
**4′**		22.3, CH_3_						

^a^ overlapping signals; ^b^ value established from the HSQC (Heteronuclear Single Quantum Coherence) spectrum; ^c^ value established from the HMBC (Heteronuclear Multiple Bond Correlation) spectrum; ^d^ measured at 100 MHz; ^e^ measured at 150 MHz.

## Data Availability

Raw data discussed in this study are available in Supplementary Materials.

## Supplementary Materials

The following supporting information can be downloaded at: https://www.mdpi.com/article/10.3390/md21100543/s1, HRMS spectra, 1D and 2D NMR spectra of all the new compounds **1–8**, population of Boltzmann-averaged conformers of compounds **1**, **2**, **7** and **8** as well as UV spectra of the isolated MAAs.
