# Supplementary material for: Isolation and Structure Elucidation of Novel Mycosporine-like Amino Acids from the Two Intertidal Red Macroalgae Bostrychia scorpioides and Catenella caespitosa
Source: Mar Drugs. 2023 Oct 18;21(10):543. doi: 10.3390/md21100543 (PMC10608480; doi:10.3390/md21100543)
Supplement: Supplementary file 1 [file marinedrugs-21-00543-s001.zip › marinedrugs-2653405-supplementary.pdf]

Supplementary Materials

Isolation and Structure Elucidation of Novel Mycosporine-like Amino Acids from the Two Intertidal Red Macroalgae *Bostrychia scorpioides* and *Catenella caespitosa*

Maria Orfanoudaki [1], Mostafa Alilou [1], Anja Hartmann [1], Julia Mayr [1], Ulf Karsten [2], Hieu Nguyen-Ngoc [1,3,4] and Markus Ganzera [1,*]

1 Institute of Pharmacy, Pharmacognosy, University of Innsbruck, Innrain 80-82, 6020 Innsbruck, Austria; orfmaria@gmail.com (M.O.); mostafa.alilou@uibk.ac.at (M.A.); anja-k-hartmann@web.de (A.H.); julia_mayr1@gmx.at (J.M.); hieu.nguyenngoc@phenikaa-uni.edu.vn (H.N.-N.)

2 Institute of Biological Sciences, Applied Ecology & Phycology, University of Rostock, Albert-Einstein-Str. 3, 18059 Rostock, Germany; ulf.karsten@uni-rostock.de

3 Faculty of Pharmacy, Phenikaa University, Hanoi 12116, Vietnam

4 A&A Green Phoenix Group JSC, Phenikaa Research and Technology Institute (PRATI), No.167 Hoang Ngan, Trung Hoa, Cau Giay, Hanoi 11313, Vietnam

* Correspondence: markus.ganzera@uibk.ac.at; Tel.: +43-512-507-58406

Contents

Figure S1. ^{1}H NMR spectrum of compound 1 in D_2O 4

Figure S2. COSY spectrum of compound 1 in D_2O 4

Figure S3. HSQC spectrum of compound 1 in D_2O 5

Figure S4. HMBC spectrum of compound 1 in D_2O 5

Figure S5. ^{13}C NMR spectrum of compound 1 in D_2O 6

Figure S6. High-resolution mass spectrum of compound 1 6

Figure S7. Overlayered conformers and population of Boltzmann averaged conformers of compound 1 optimized at the DFT/wb97xd/6-31+g(d,p) level in the gas phase. 6

Figure S8. ^{1}H NMR spectrum of compound 2 in D_2O 7

Figure S9. COSY spectrum of compound 2 in D_2O 7

Figure S10. HSQC spectrum of compound 2 in D_2O 8

Figure S11. HMBC spectrum of compound 2 in D_2O 8

Figure S12. ^{13}C NMR spectrum of compound 2 in D_2O 9

Figure S13. High-resolution mass spectrum of compound 2 9

Figure S14. Overlayered conformers and population of Boltzmann averaged conformers of compound 2 optimized at the DFT/wb97xd/6-31+g(d,p) level in the gas phase. 10

Figure S15. ^{1}H NMR spectrum of compounds 3 and 4 in D_2O 10

Figure S16. COSY spectrum of compounds 3 and 4 in D_2O 11

Figure S17. HSQC spectrum of compounds 3 and 4 in D_2O 12

Figure S18. HMBC spectrum of compounds 3 and 4 in D_2O 12

Figure S19. ^{13}C NMR spectrum of compounds 3 and 4 in D_2O 13

Figure S20. High-resolution mass spectrum of compounds 3 and 4 13

Figure S21. ^{1}H NMR spectrum of compounds 5 and 6 in D_2O 14

Figure S22. COSY spectrum of compounds 5 and 6 in D_2O 14

Figure S23. HSQC spectrum of compounds 5 and 6 in D_2O 15

Figure S24. HMBC spectrum of compounds 5 and 6 in D_2O 16

Figure S25. ^{13}C NMR spectrum of compounds 5 and 6 in D_2O 16

Figure S26. High-resolution mass spectrum of compounds 5 and 6 16

Figure S27. ^{1}H NMR spectrum of compound 7 in D_2O 17

Figure S28. COSY spectrum of compound 7 in D_2O 17

Figure S29. HSQC spectrum of compound 7 in D_2O 18

Figure S30. HMBC spectrum of compound 7 in D_2O 18

Figure S31. ^{13}C NMR spectrum of compound 7 in D_2O 19

Figure S32. High-resolution mass spectrum of compound 7 19

Figure S33. Overlayered conformers and population of Boltzmann averaged conformers of compound 7 optimized at the DFT/wb97xd/6-31+g(d,p) level in the gas phase. 20

Figure S34. ^{1}H NMR spectrum of compound 8 in D_2O 21

Figure S35. COSY spectrum of compound 8 in D_2O........ 21

Figure S36. HSQC spectrum of compound 8 in D_2O........ 22

Figure S37. HMBC spectrum of compound 8 in D_2O........ 22

Figure S38. ^{13}C NMR spectrum of compound 8 in D_2O 23

Figure S39. High-resolution mass spectrum of compound 8 23

Figure S40. Overlayered conformers and population of Boltzmann averaged conformers of compound 8 optimized at the DFT/wb97xd/6-31+g(d,p) level in the gas phase. 23

Figure S41. Absorbance spectra of compounds 1-8 in water. 24

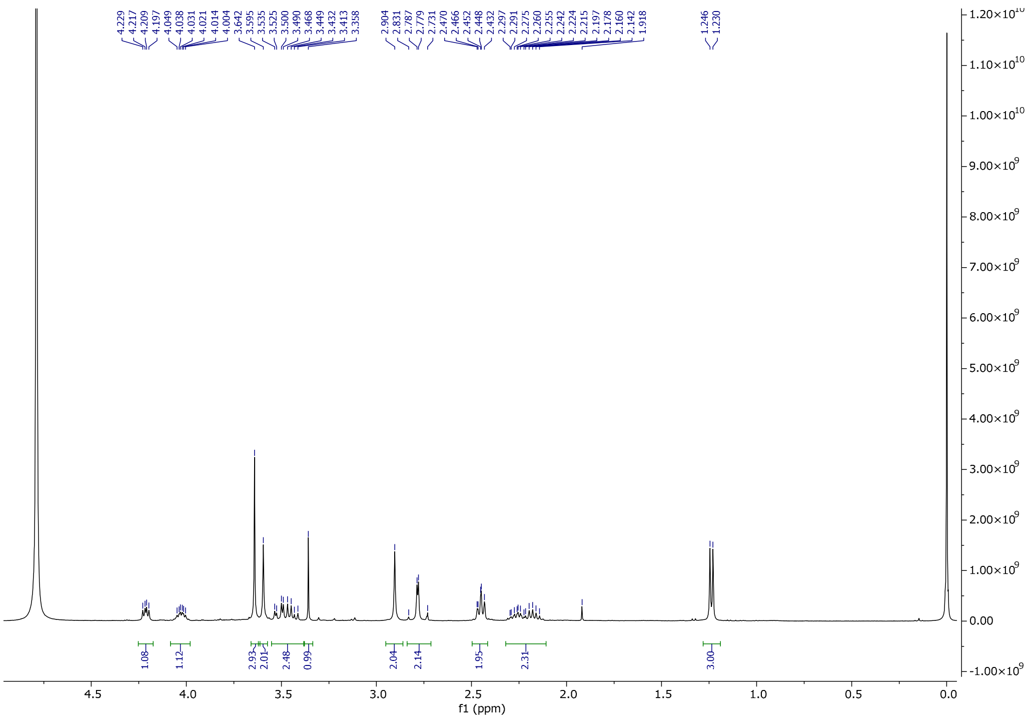

Figure S1. ^{1}H NMR spectrum of compound **1** in D_2O

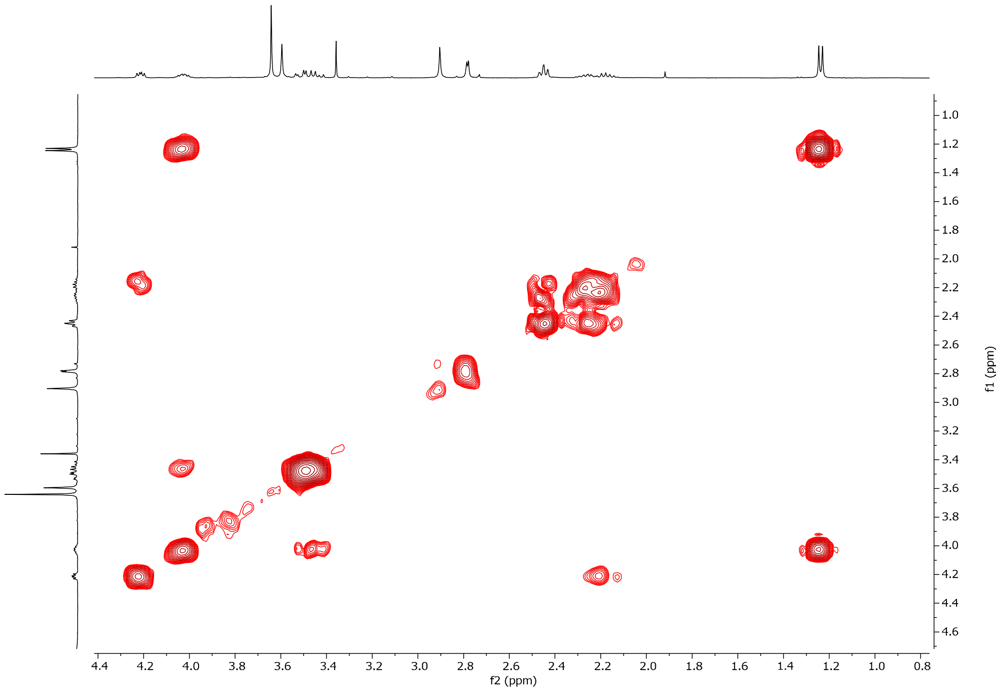

Figure S2. COSY spectrum of compound **1** in D_2O

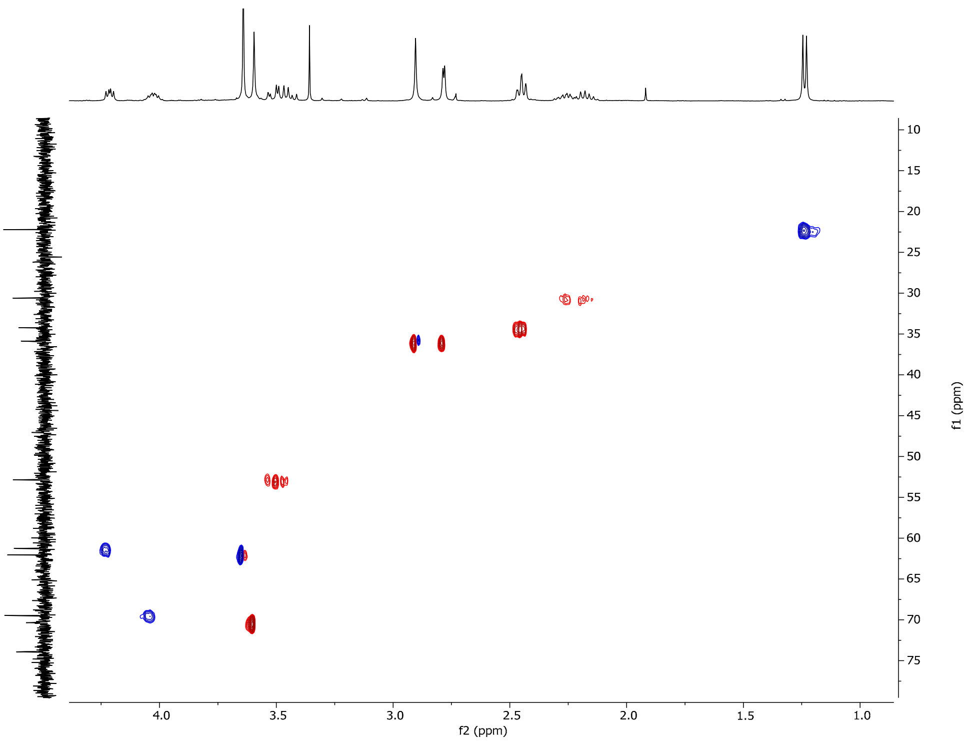

Figure S3. HSQC spectrum of compound **1** in D_2O

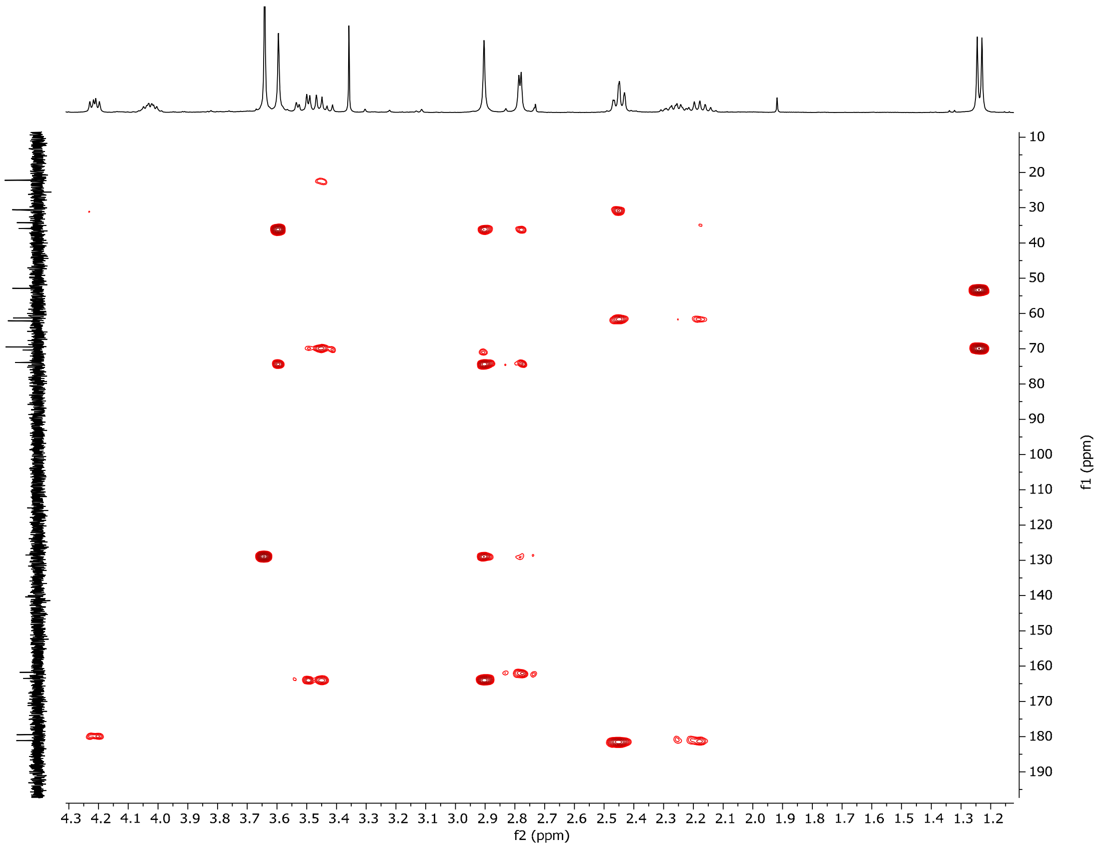

Figure S4. HMBC spectrum of compound **1** in D_2O

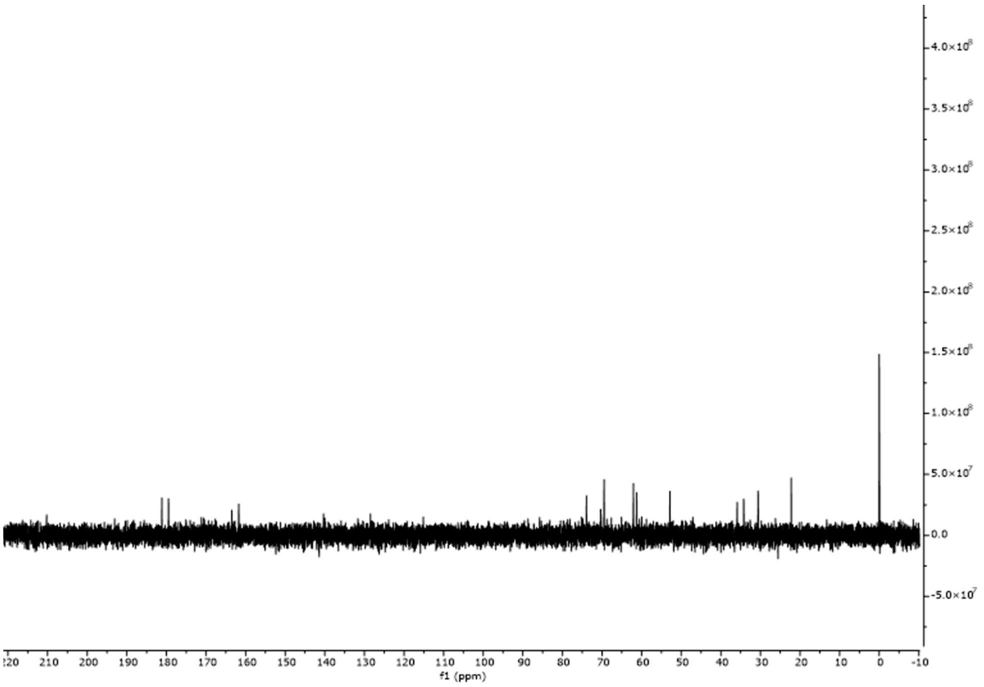

Figure S5. ^{13}C NMR spectrum of compound **1** in D_2O

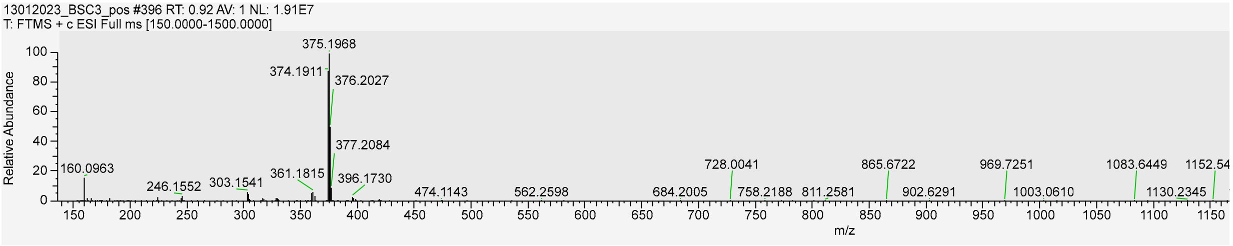

Figure S6. High-resolution mass spectrum of compound **1**

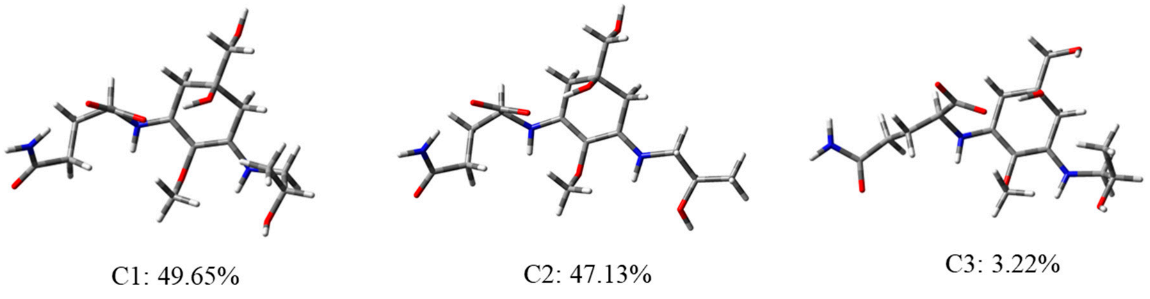

Figure S7. Overlayered conformers and population of Boltzmann averaged conformers of compound **1** optimized at the DFT/wb97xd/6-31+g(d,p) level in the gas phase.

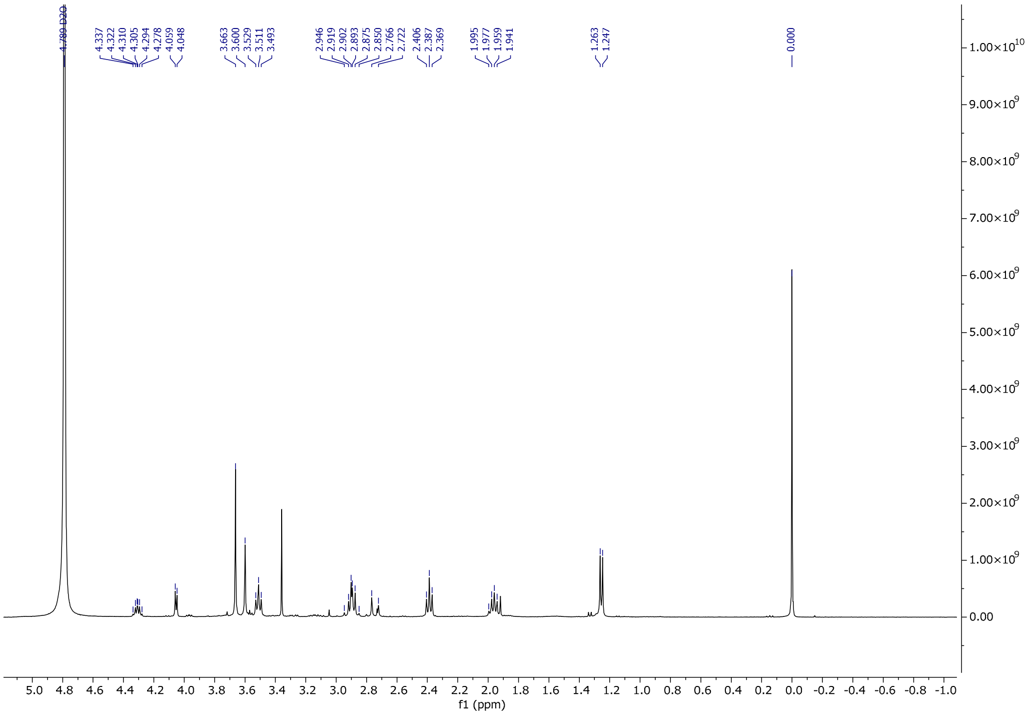

Figure S8. ^{1}H NMR spectrum of compound **2** in D_2O

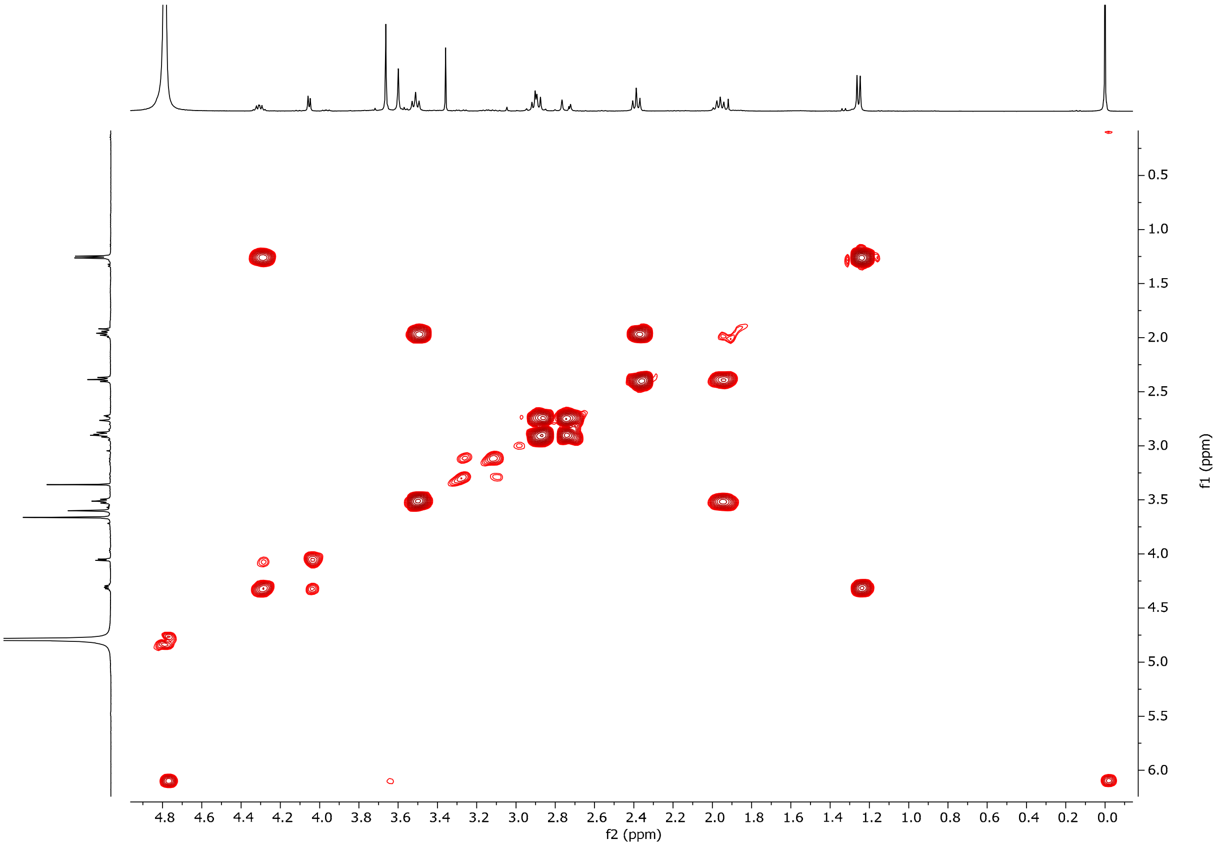

Figure S9. COSY spectrum of compound **2** in D_2O

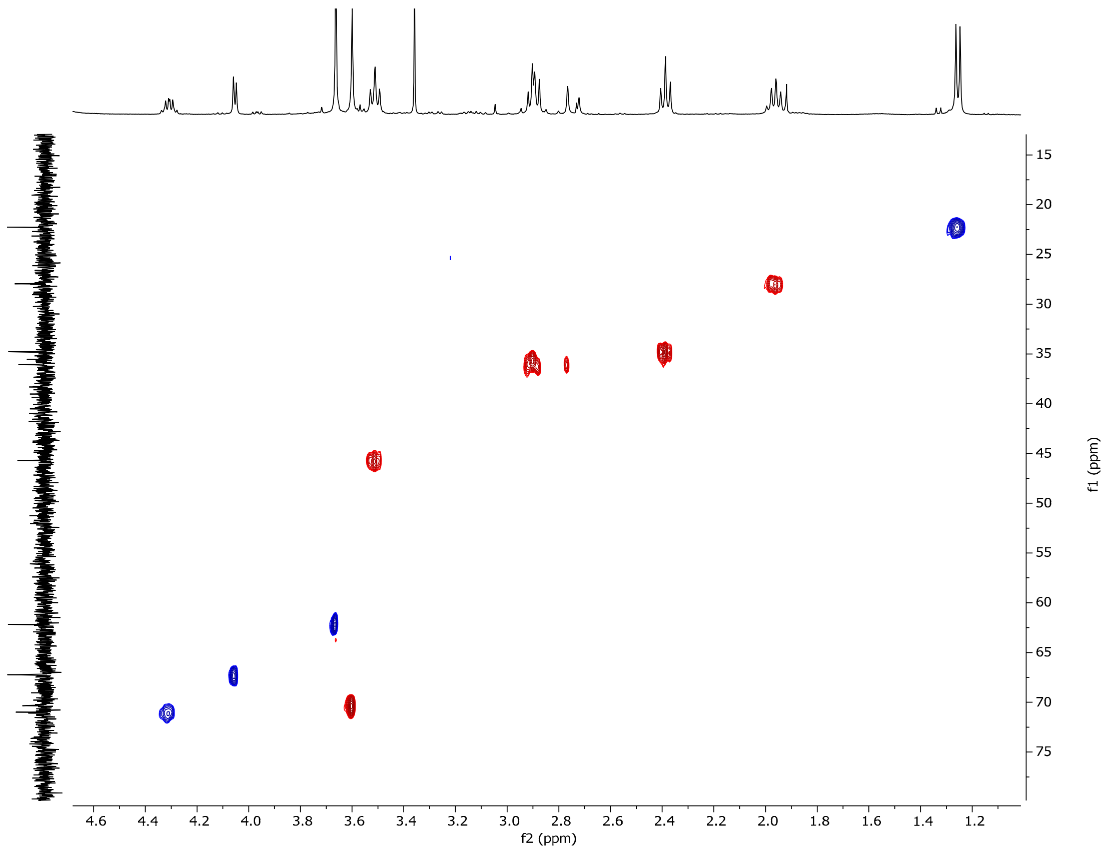

Figure S10. HSQC spectrum of compound **2** in D_2O

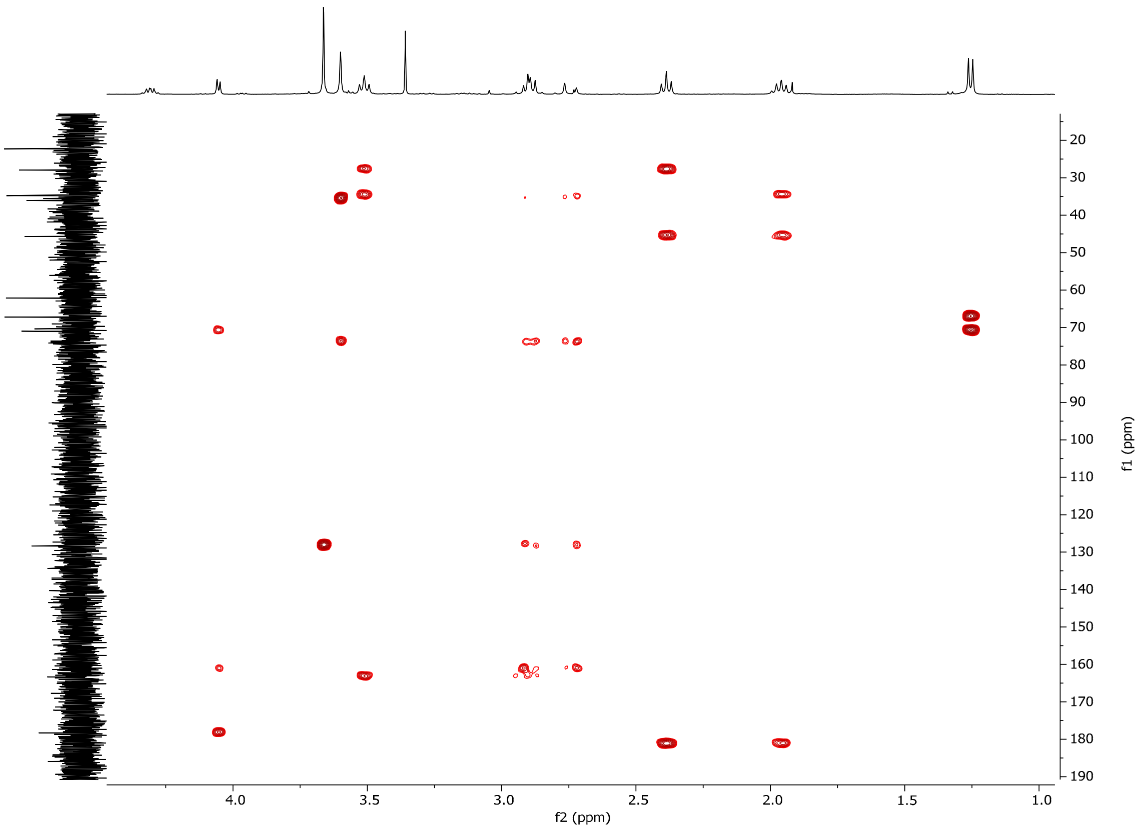

Figure S11. HMBC spectrum of compound **2** in D_2O

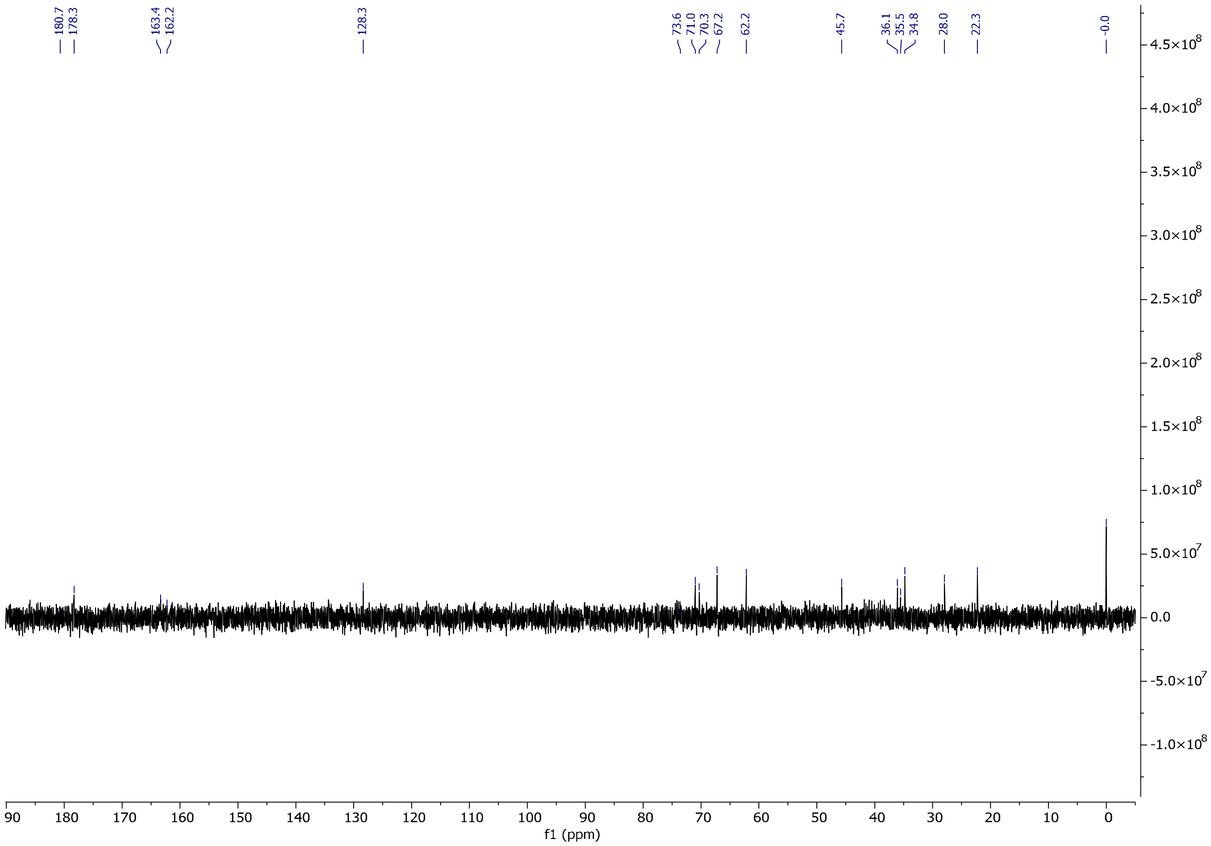

Figure S12. ^{13}C NMR spectrum of compound **2** in D_2O

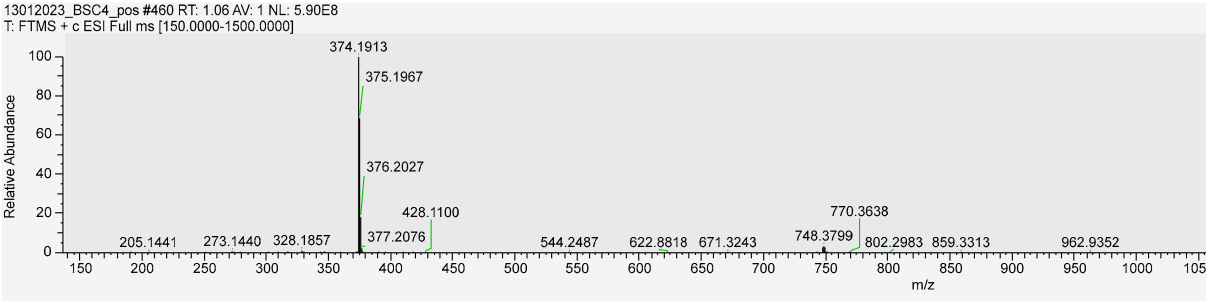

Figure S13. High-resolution mass spectrum of compound **2**

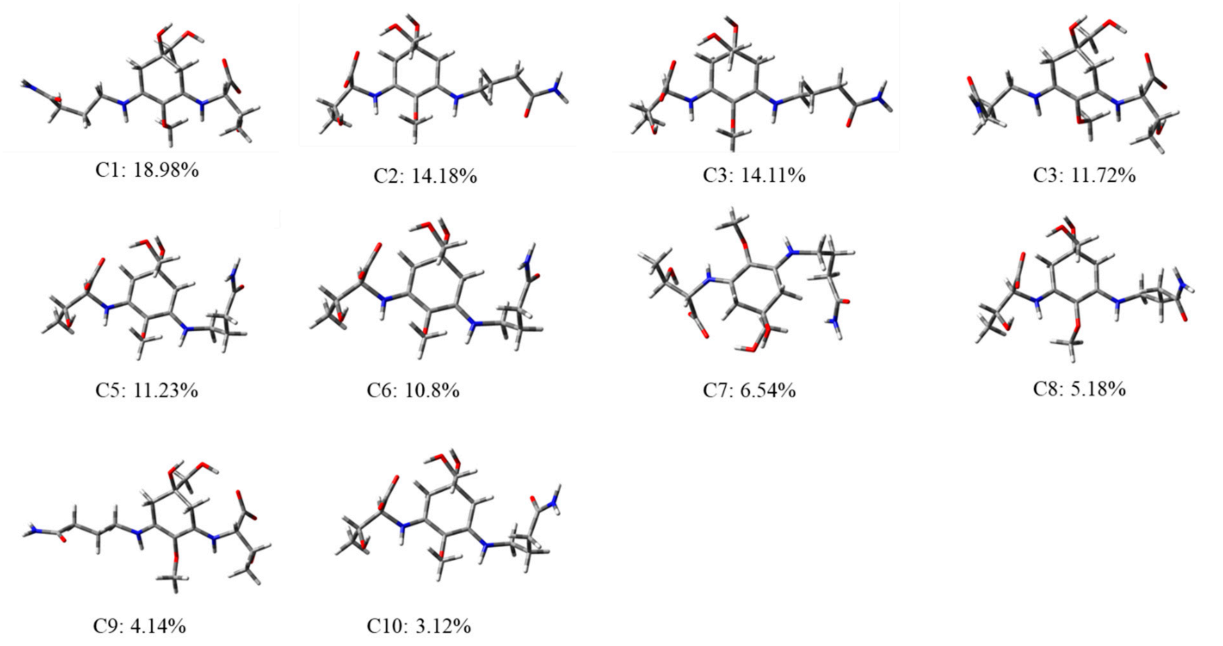

Figure S14. Overlayered conformers and population of Boltzmann averaged conformers of compound **2** optimized at the DFT/wb97xd/6-31+g(d,p) level in the gas phase.

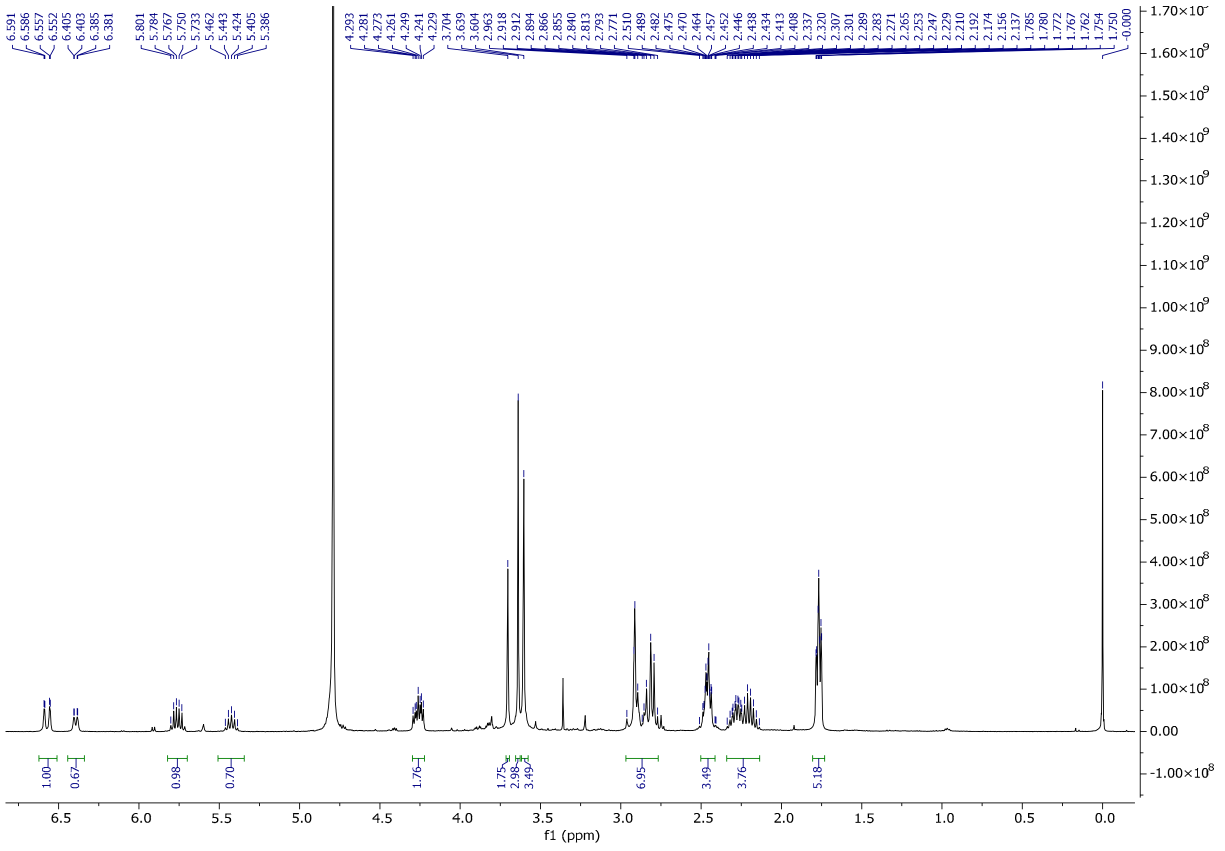

Figure S15. ^{1}H NMR spectrum of compounds **3** and **4** in D_2O

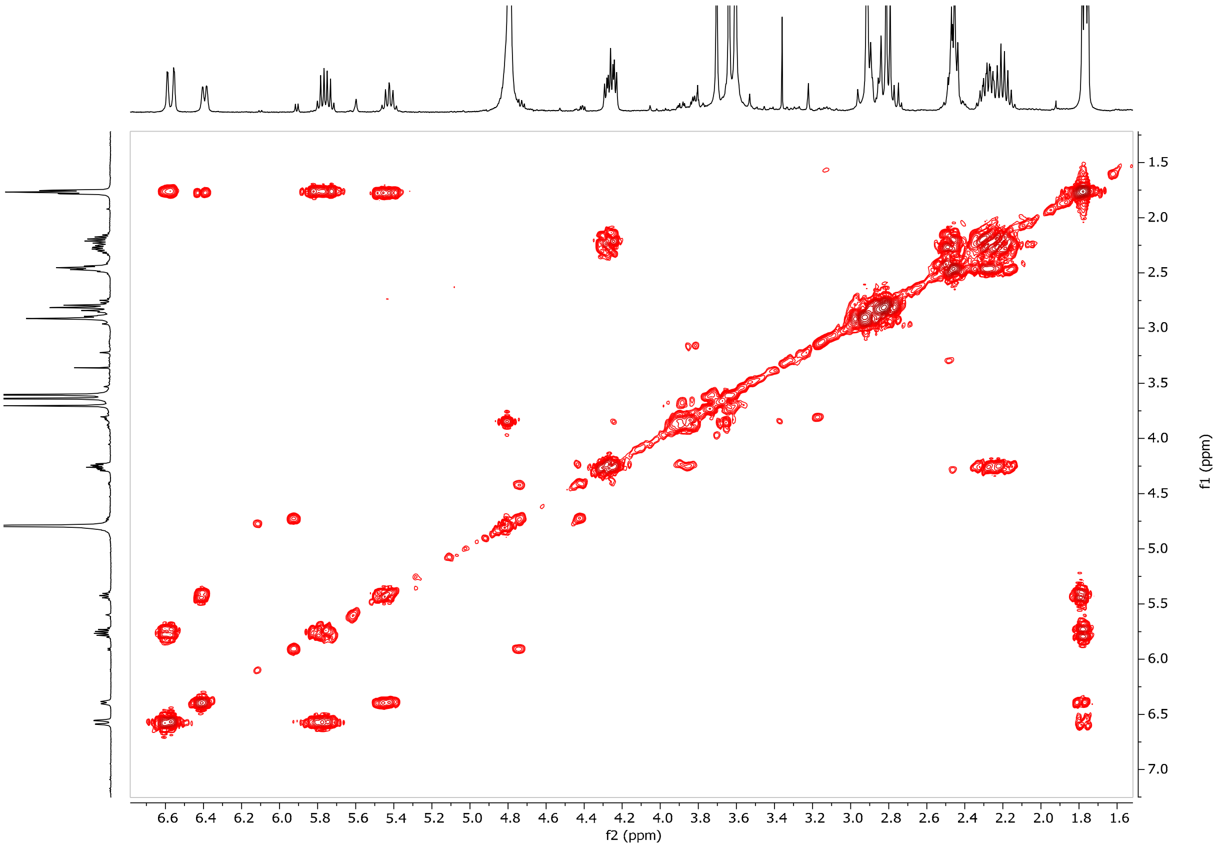

Figure S16. COSY spectrum of compounds **3** and **4** in D_2O

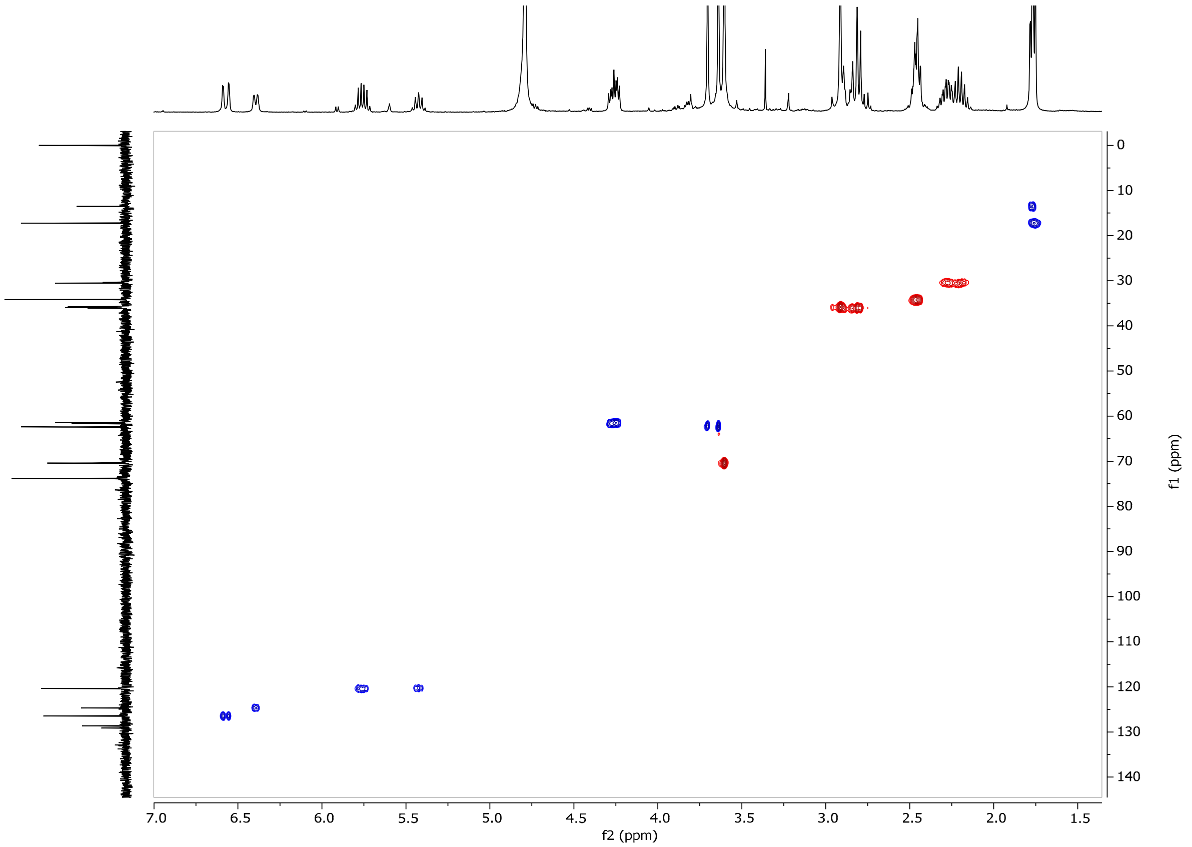

Figure S17. HSQC spectrum of compounds **3** and **4** in D_2O

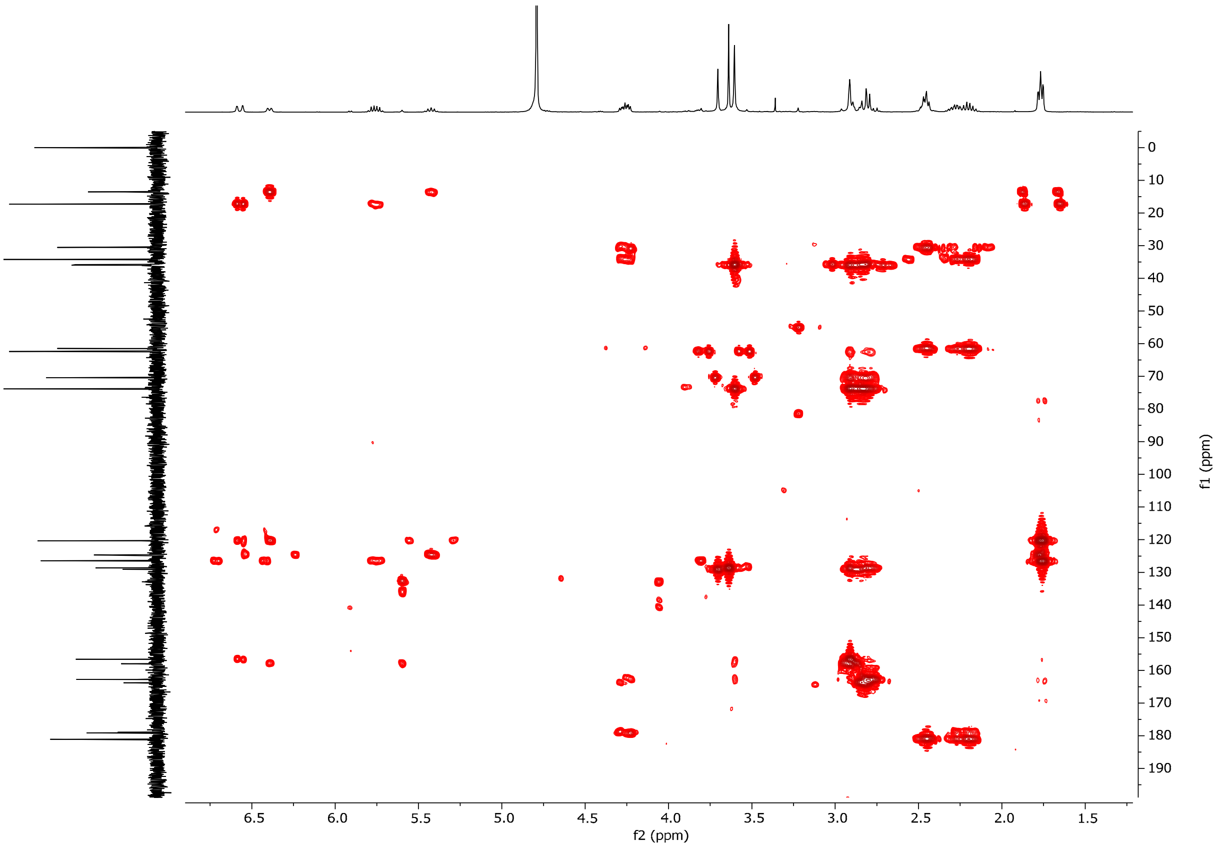

Figure S18. HMBC spectrum of compounds **3** and **4** in D_2O

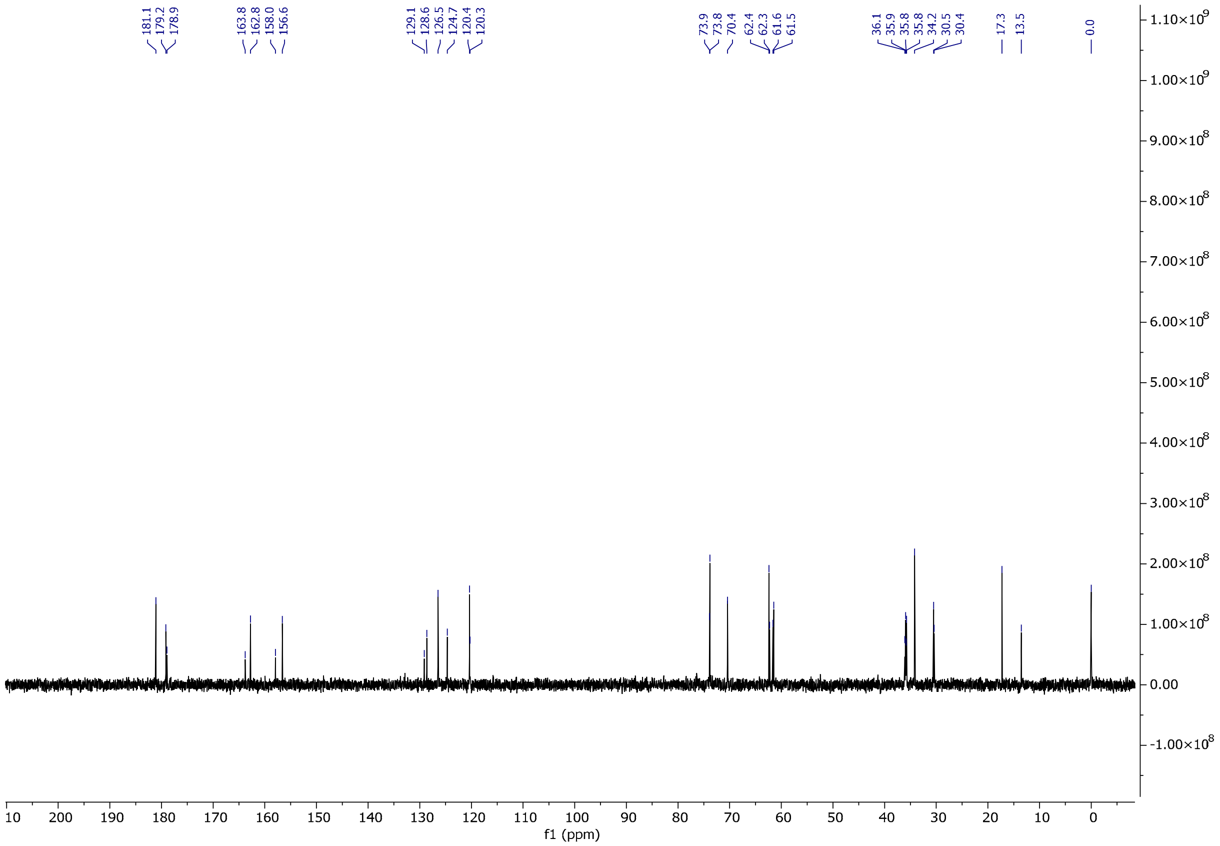

Figure S19. ^{13}C NMR spectrum of compounds **3** and **4** in D_2O

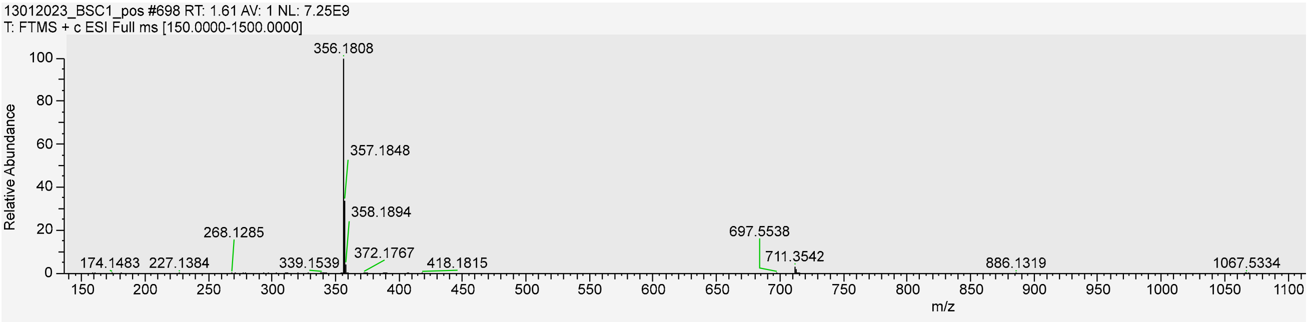

Figure S20. High-resolution mass spectrum of compounds **3** and **4**

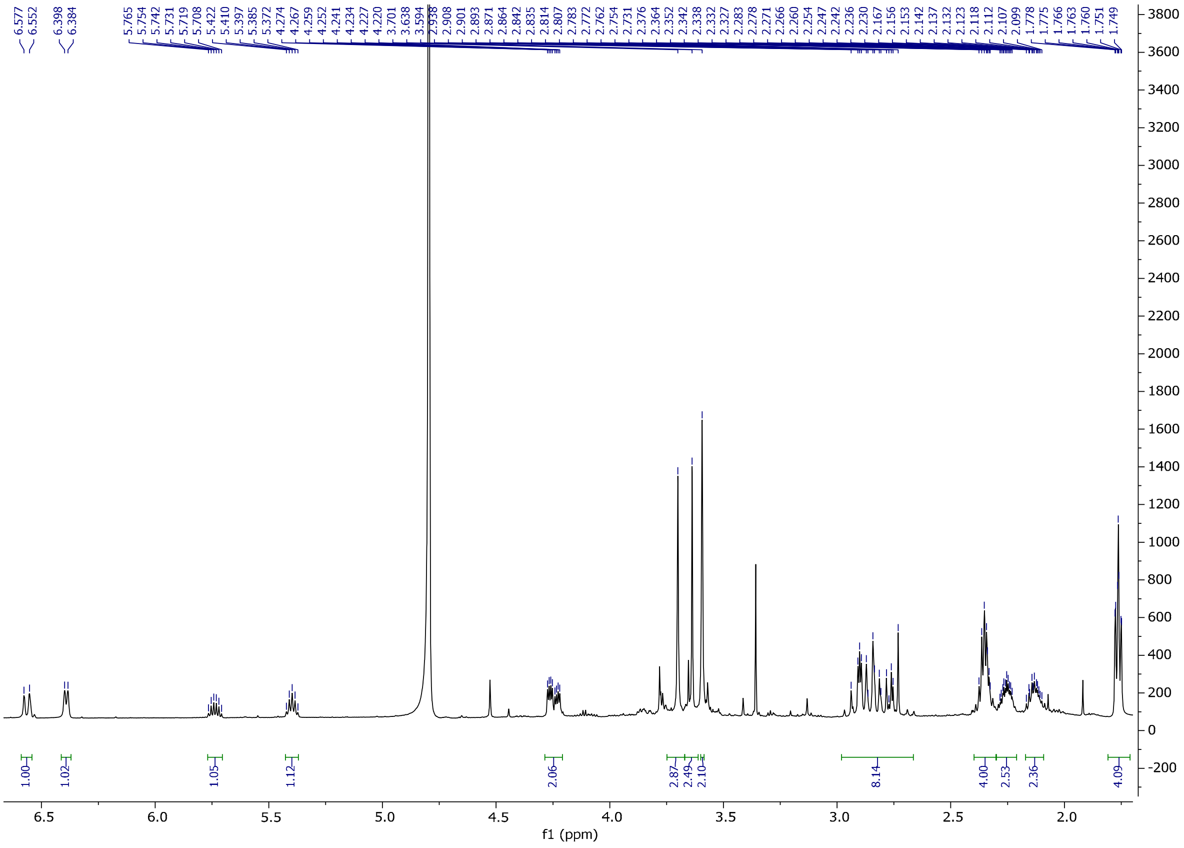

Figure S21. ^{1}H NMR spectrum of compounds **5** and **6** in D_2O

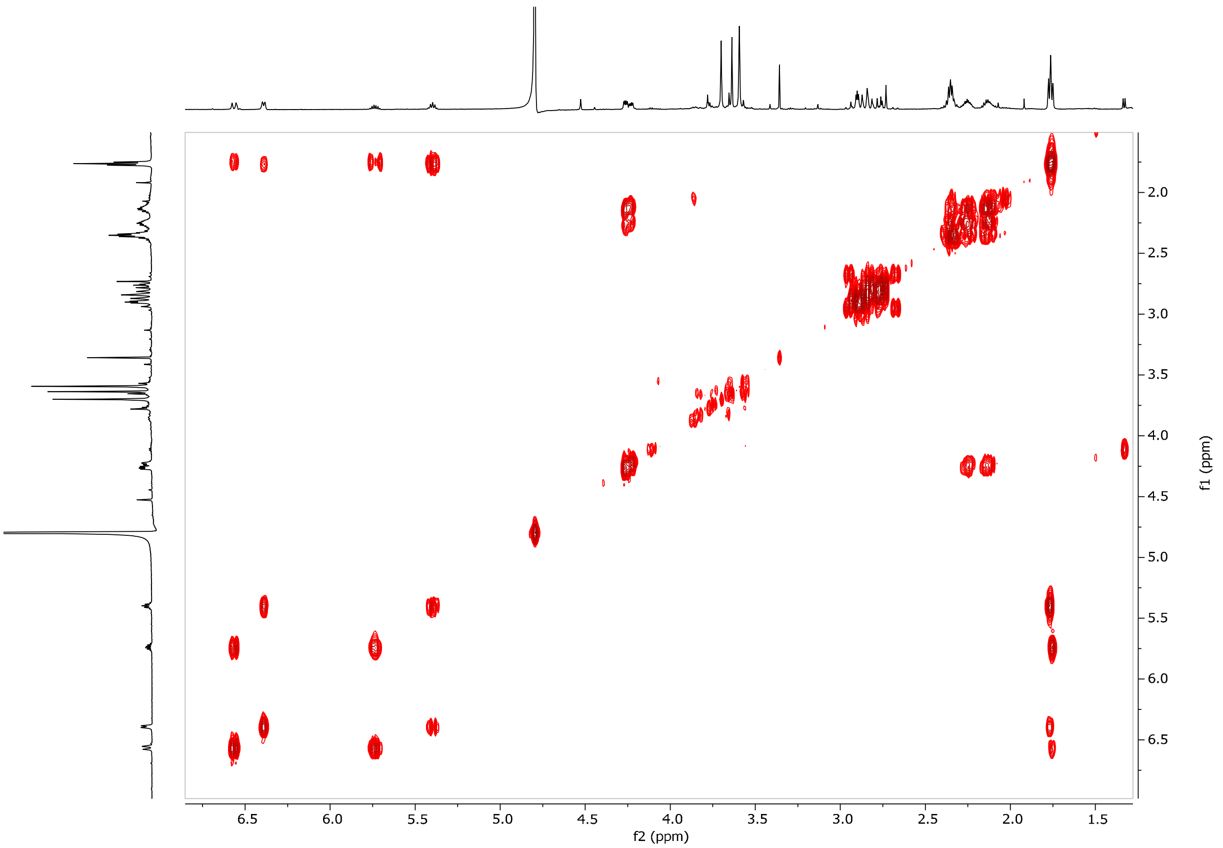

Figure S22. COSY spectrum of compounds **5** and **6** in D_2O

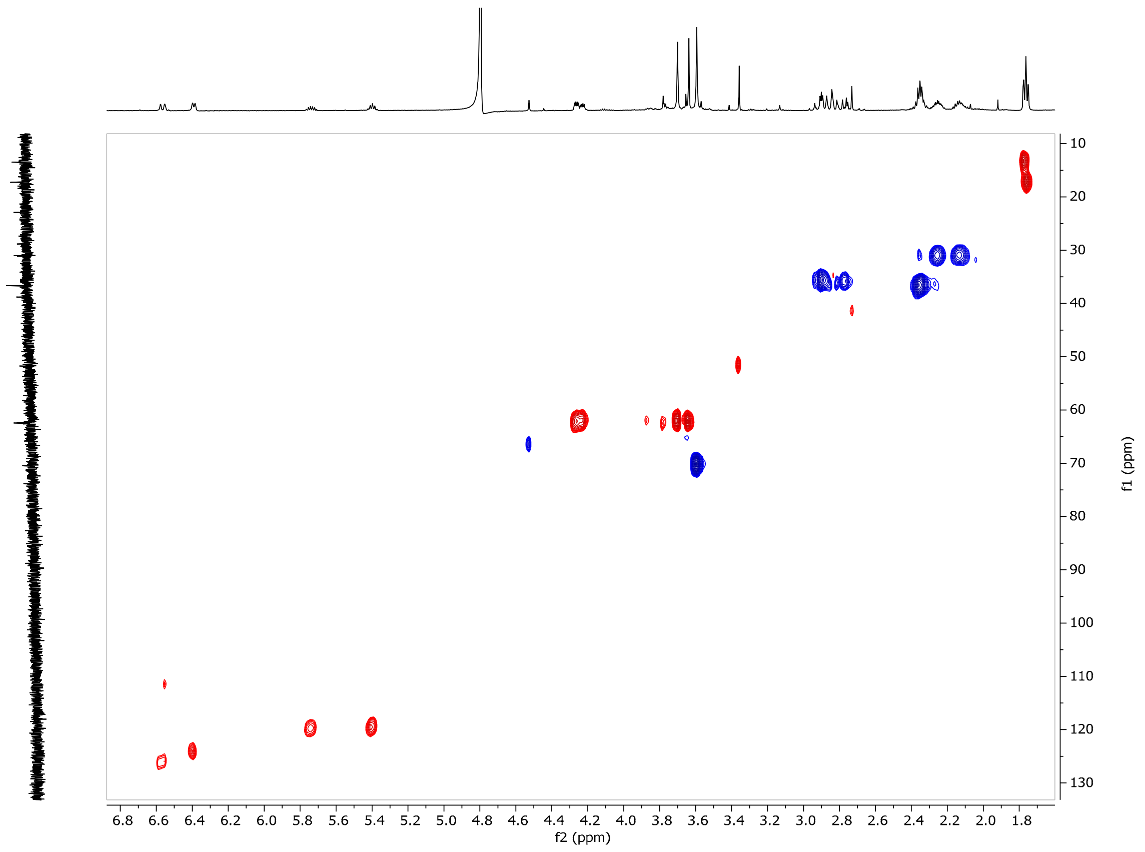

Figure S23. HSQC spectrum of compounds **5** and **6** in D_2O

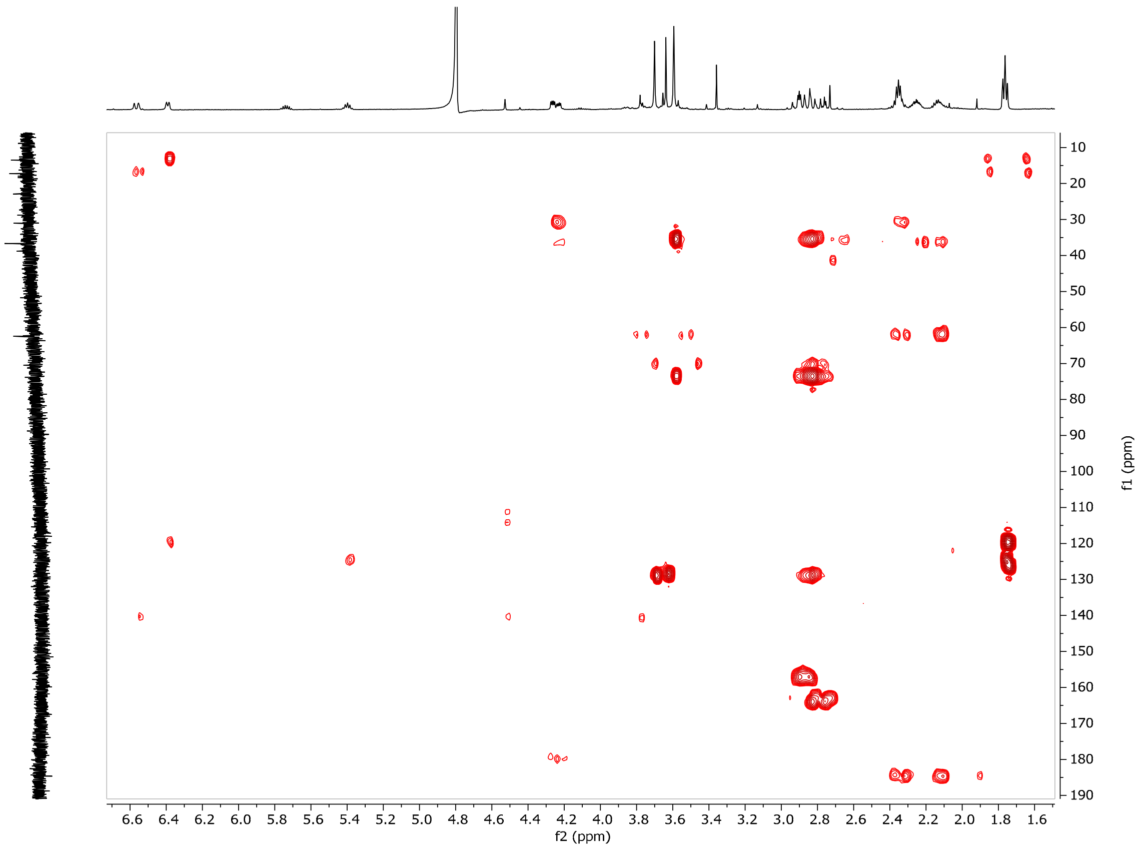

Figure S24. HMBC spectrum of compounds **5** and **6** in D_2O

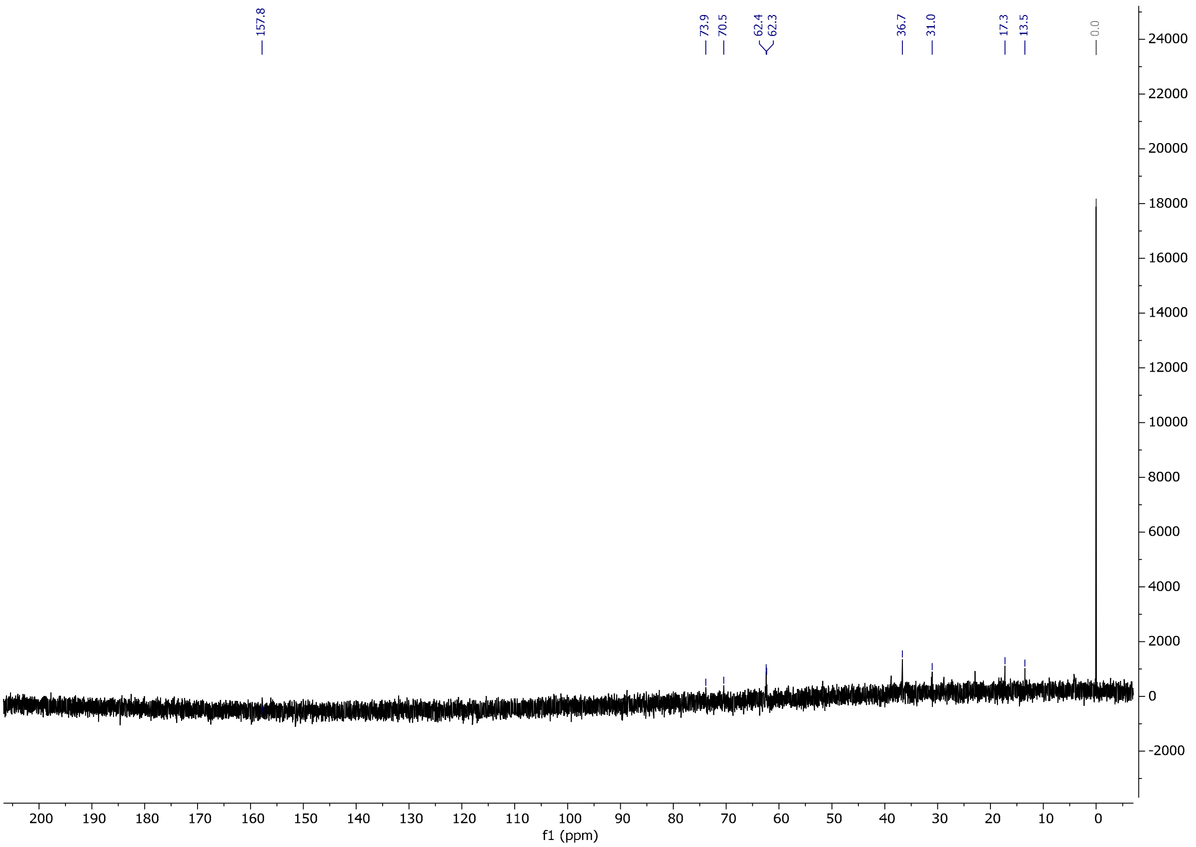

Figure S25. ^{13}C NMR spectrum of compounds **5** and **6** in D_2O

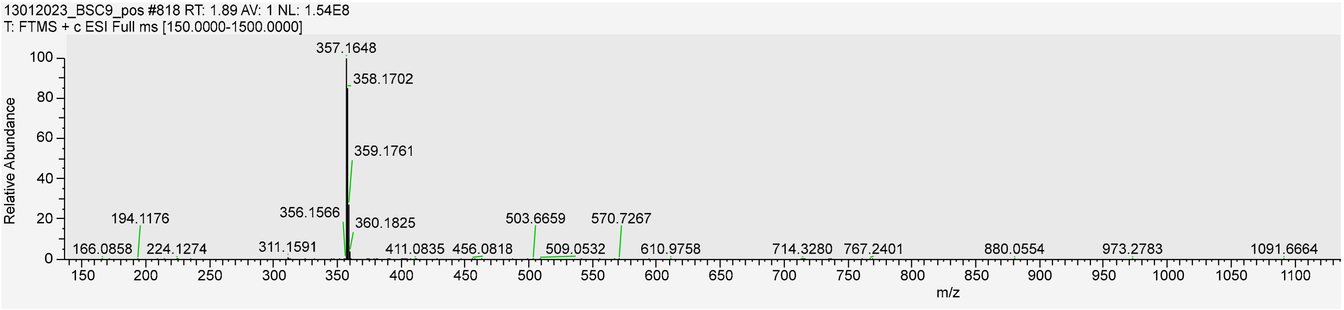

Figure S26. High-resolution mass spectrum of compounds **5** and **6**

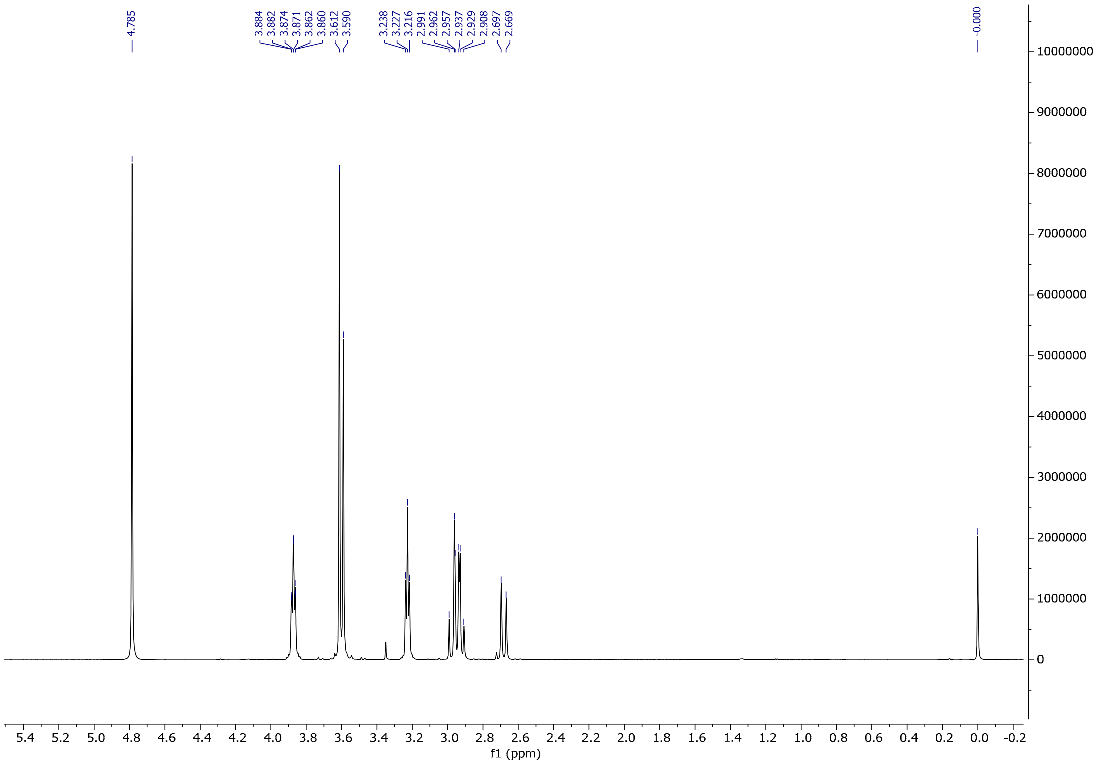

Figure S27. ^{1}H NMR spectrum of compound **7** in D_2O

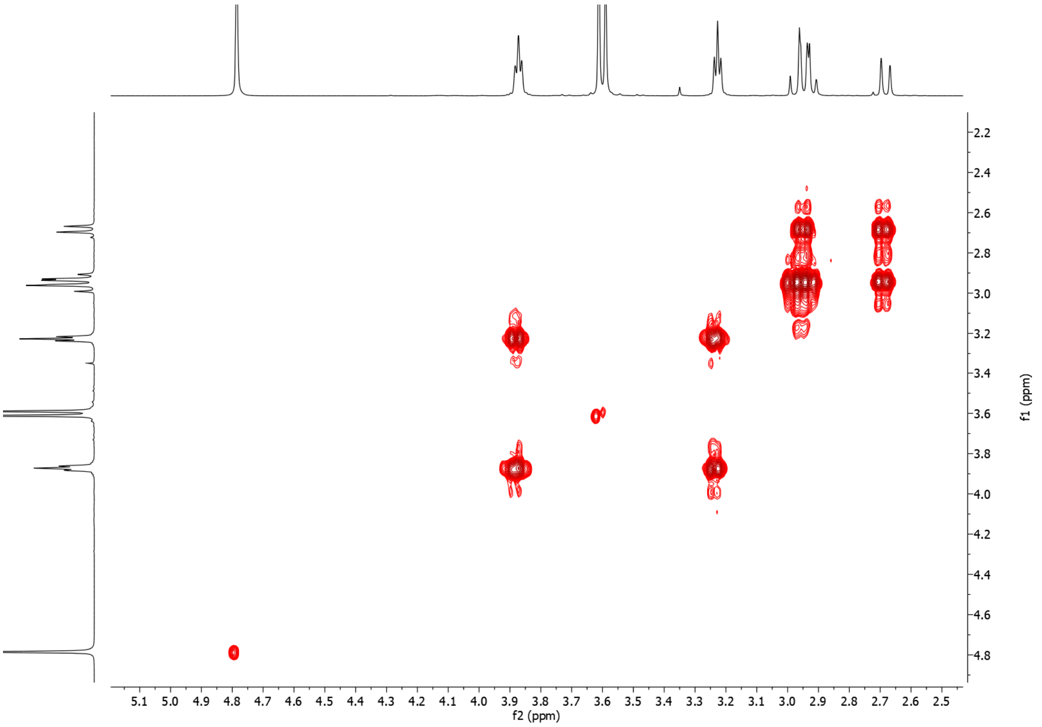

Figure S28. COSY spectrum of compound **7** in D_2O

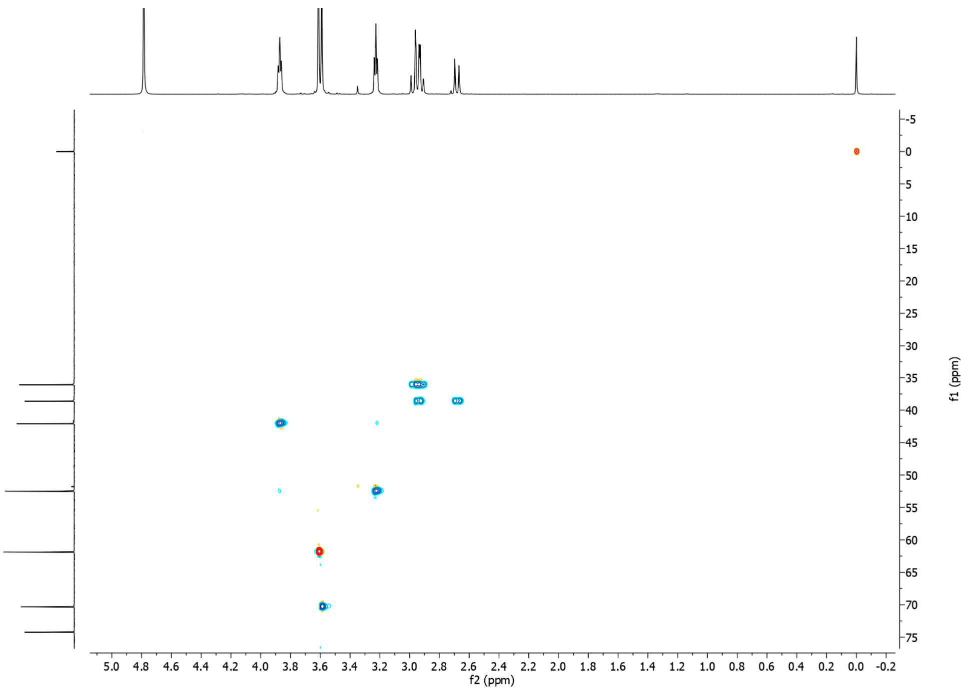

Figure S29. HSQC spectrum of compound **7** in D_2O

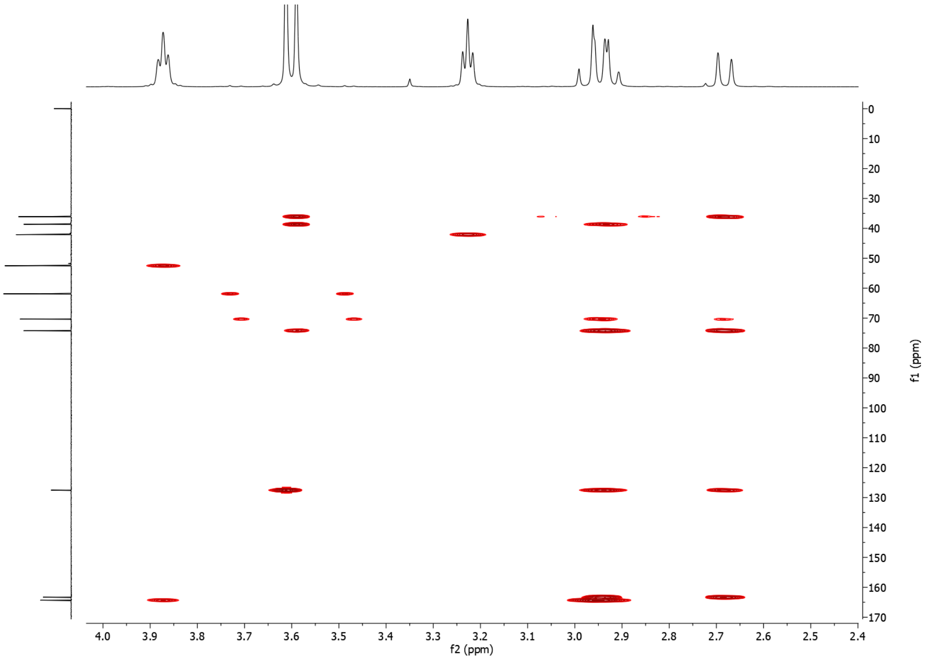

Figure S30. HMBC spectrum of compound **7** in D_2O

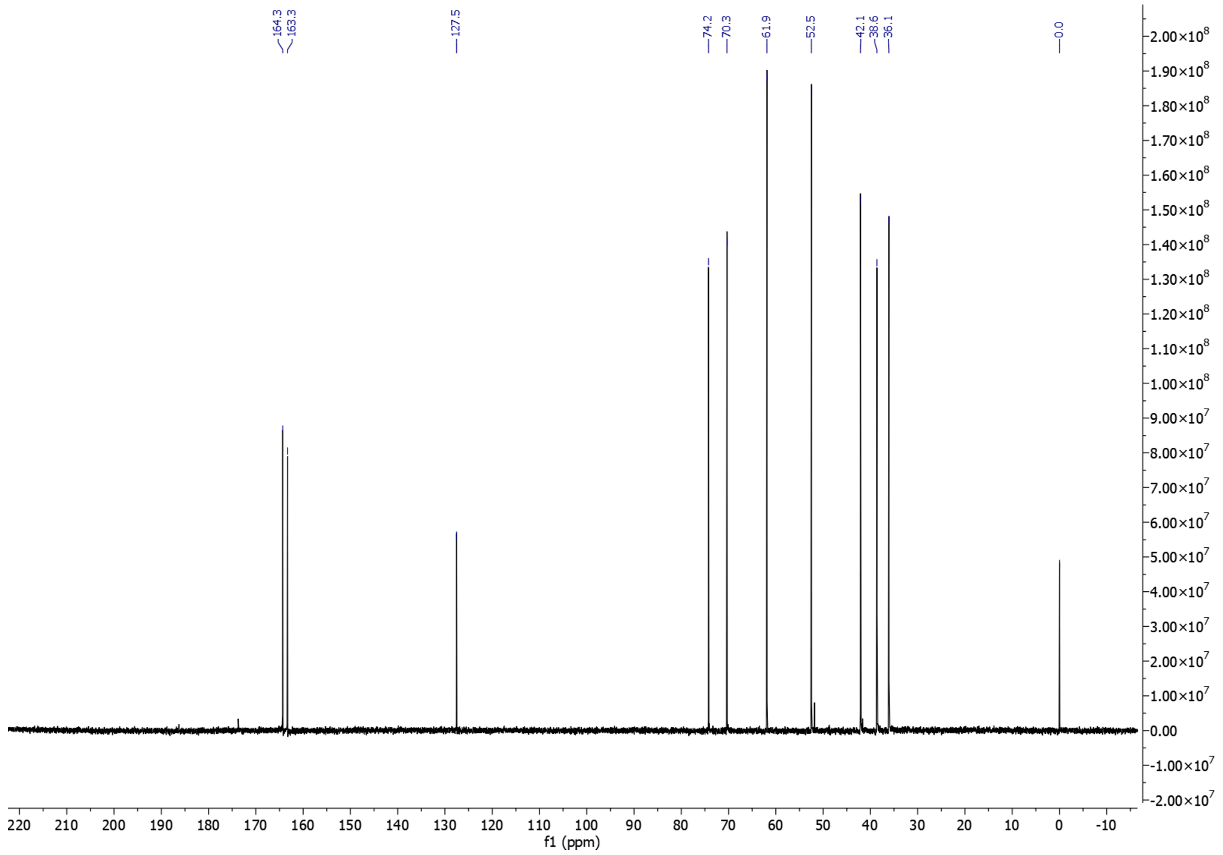

Figure S31. ^{13}C NMR spectrum of compound **7** in D_2O

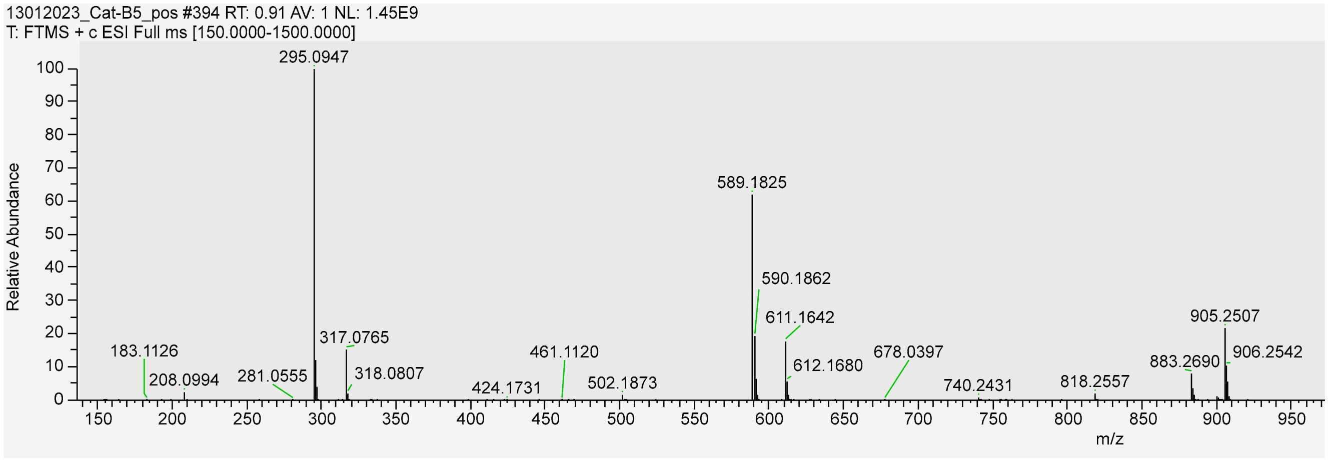

Figure S32. High-resolution mass spectrum of compound **7**

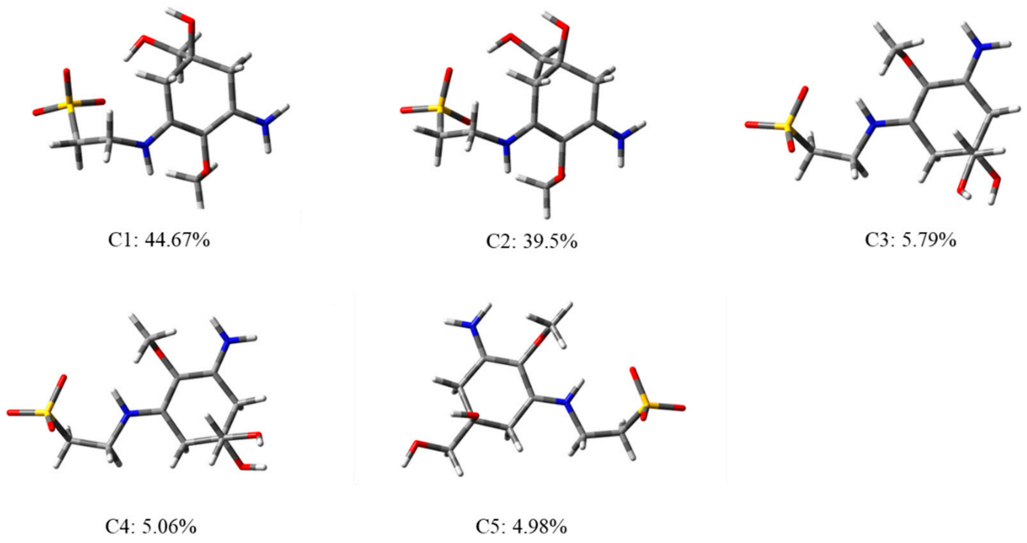

Figure S33. Overlayered conformers and population of Boltzmann averaged conformers of compound **7** optimized at the DFT/wb97xd/6-31+g(d,p) level in the gas phase.

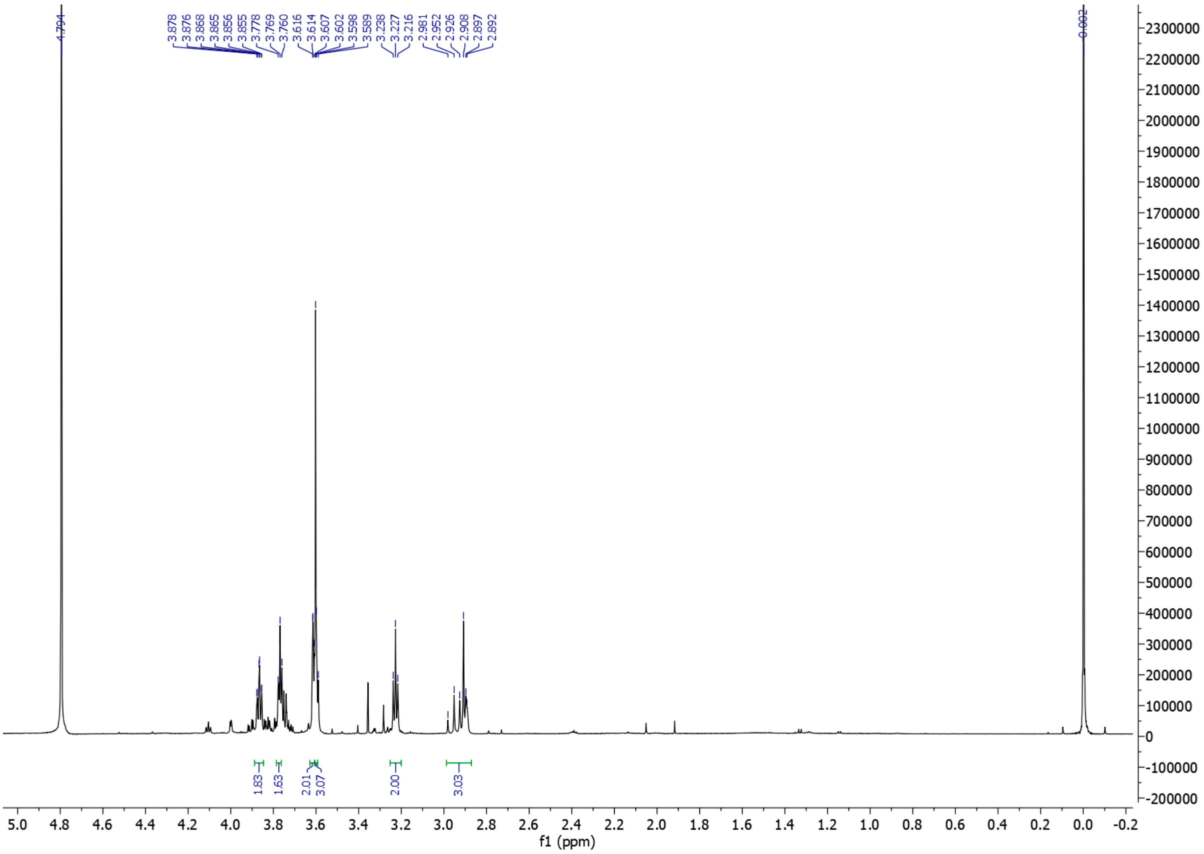

Figure S34. ^{1}H NMR spectrum of compound **8** in D_2O

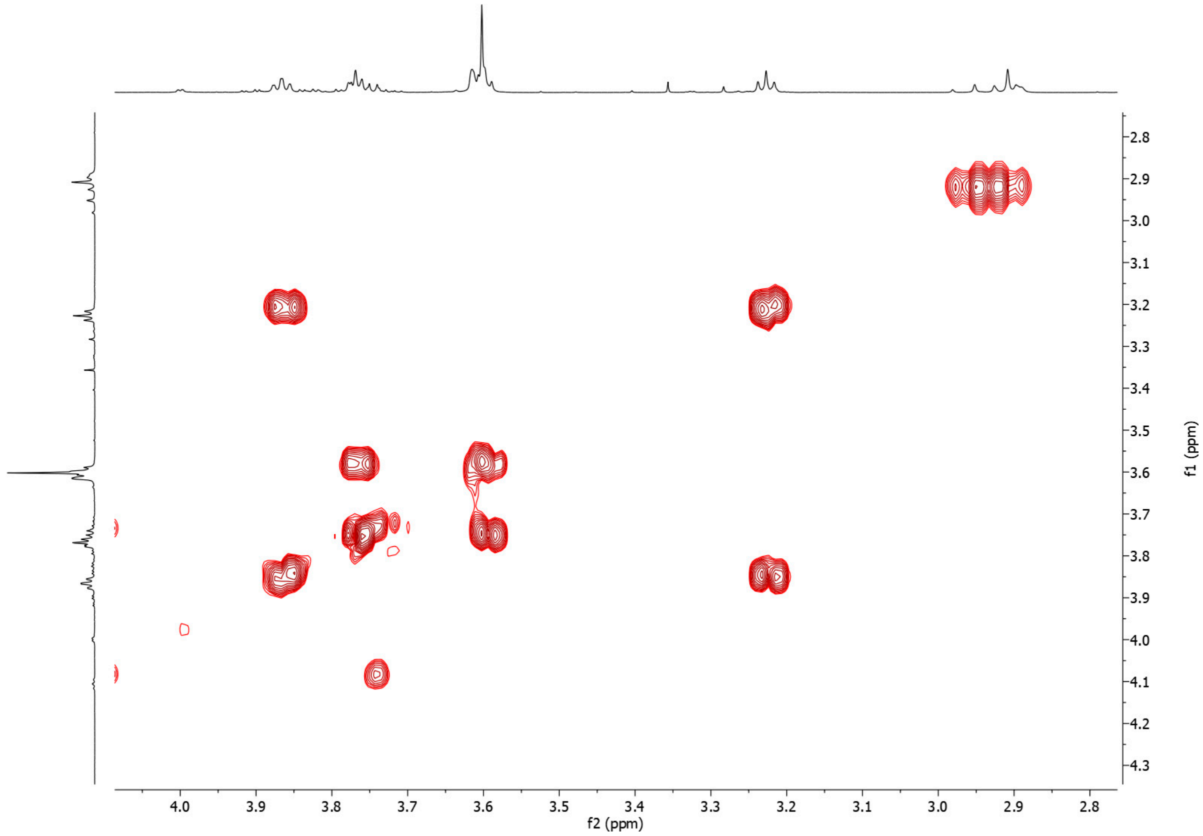

Figure S35. COSY spectrum of compound **8** in D_2O

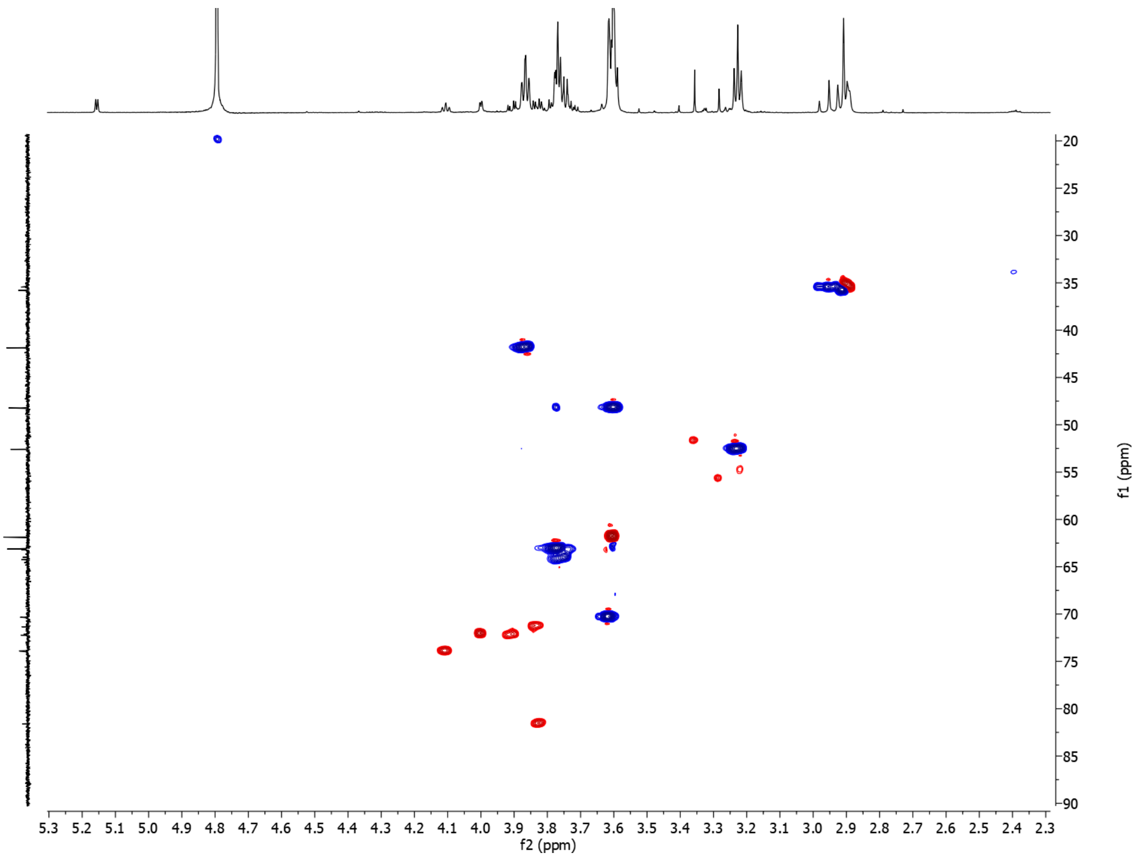

Figure S36. HSQC spectrum of compound **8** in D_2O

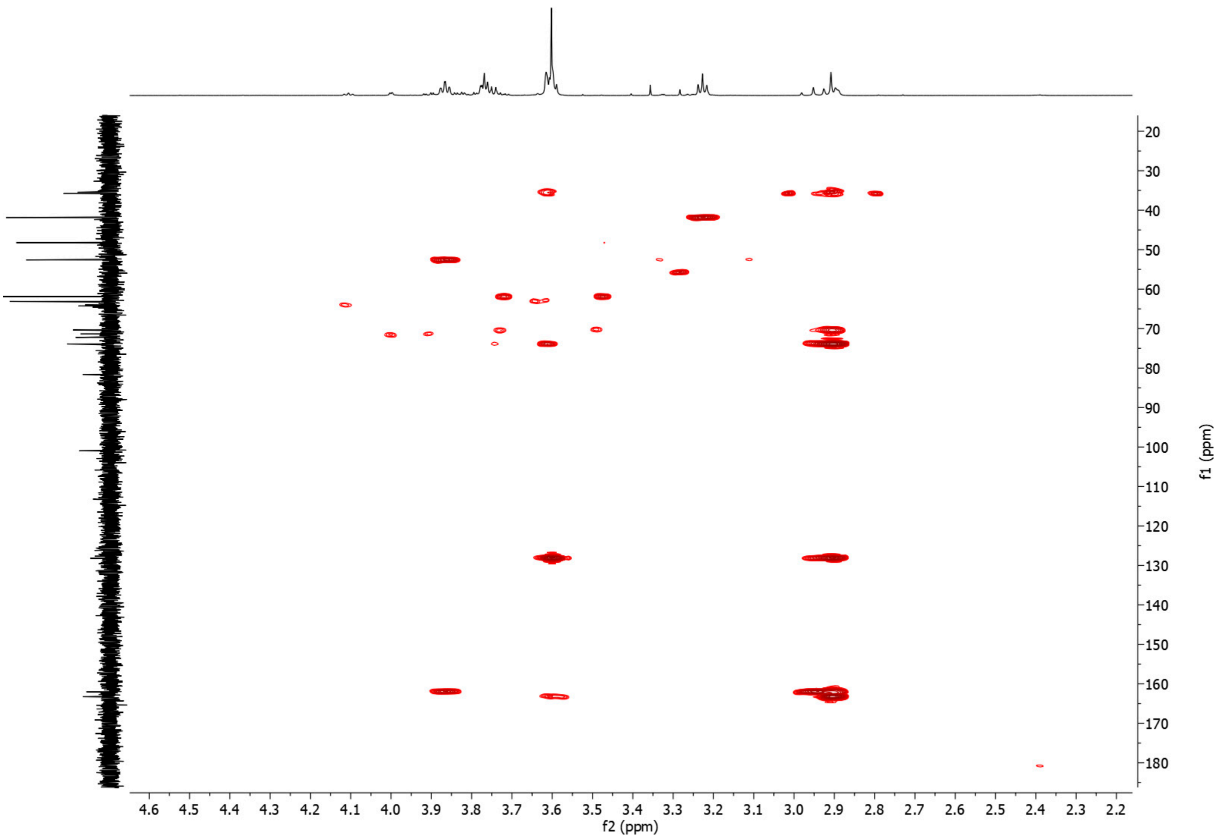

Figure S37. HMBC spectrum of compound **8** in D_2O

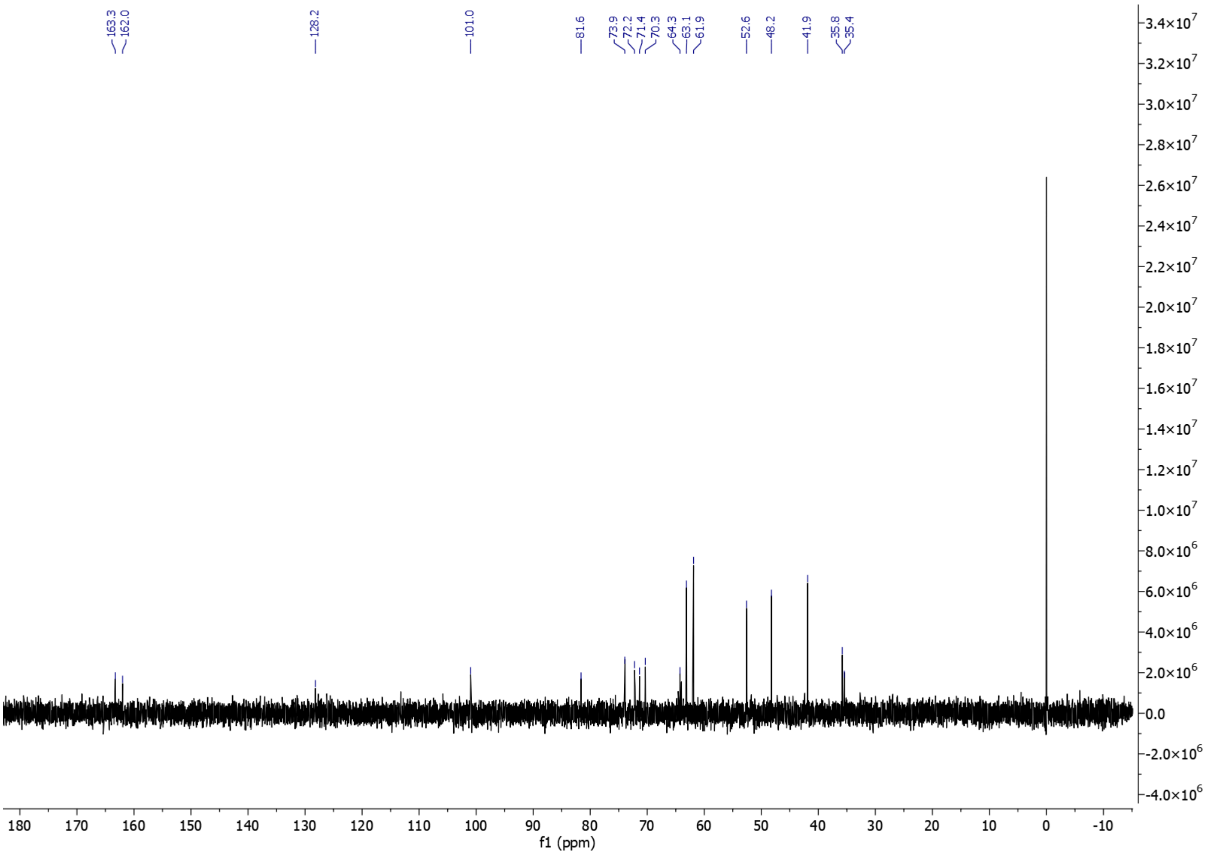

Figure S38. ^{13}C NMR spectrum of compound **8** in D_2O

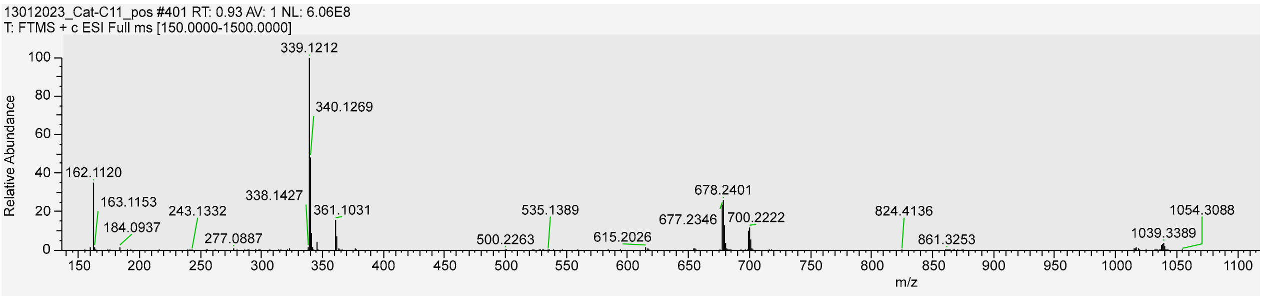

Figure S39. High-resolution mass spectrum of compound **8**

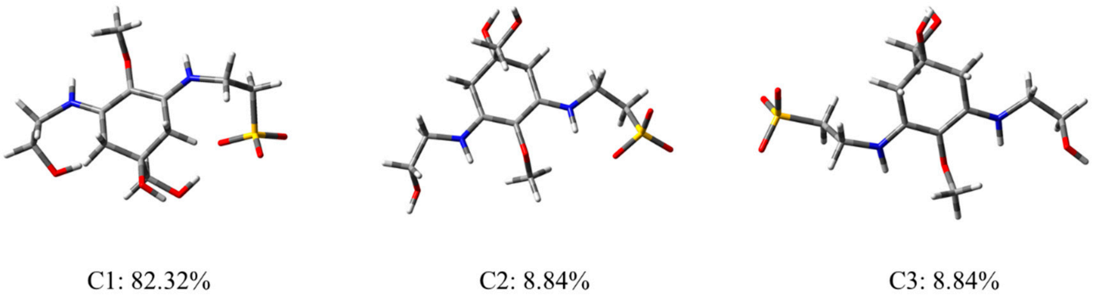

Figure S40. Overlayered conformers and population of Boltzmann averaged conformers of compound **8** optimized at the DFT/wb97xd/6-31+g(d,p) level in the gas phase.

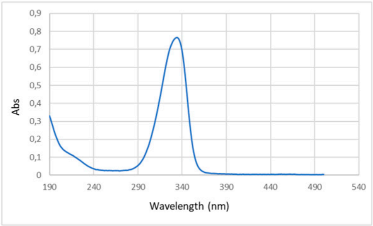

Compound **1**

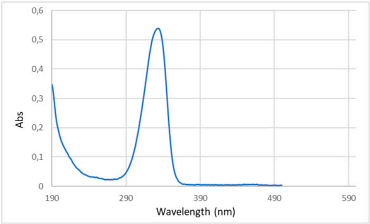

Compound **2**

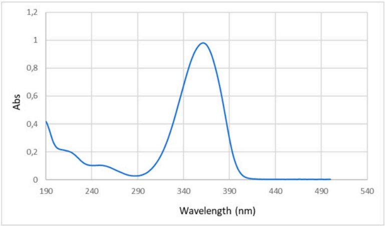

Compounds **3** and **4**

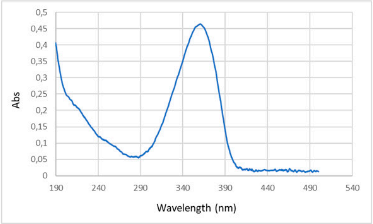

Compounds **5** and **6**

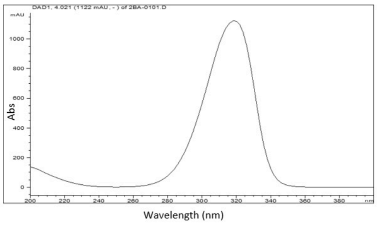

Compound **7**

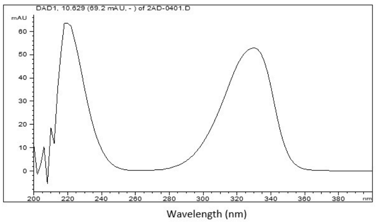

Compound **8**

Figure S41. Absorbance spectra of compounds **1-8** in water.
